# Ekman-driven salt transport as a key mechanism for open-ocean polynya formation at Maud Rise

**DOI:** 10.1126/sciadv.adj0777

**Published:** 2024-05-01

**Authors:** Aditya Narayanan, Fabien Roquet, Sarah T. Gille, Birte Gülk, Matthew R. Mazloff, Alessandro Silvano, Alberto C. Naveira Garabato

**Affiliations:** ^1^ Department of Marine Science, University of Gothenburg, Gothenburg, Sweden.; ^2^ Ocean and Earth Science, University of Southampton, National Oceanography Centre, Southampton, UK.; ^3^ Scripps Institution of Oceanography, University of California, San Diego, San Diego, CA, USA.

## Abstract

Open-ocean polynyas formed over the Maud Rise, in the Weddell Sea, during the winters of 2016–2017. Such polynyas are rare events in the Southern Ocean and are associated with deep convection, affecting regional carbon and heat budgets. Using an ocean state estimate, we found that during 2017, early sea ice melting occurred in response to enhanced vertical mixing of heat, which was accompanied by mixing of salt. The melting sea ice compensated for the vertically mixed salt, resulting in a net buoyancy gain. An additional salt input was then necessary to destabilize the upper ocean. This came from a hitherto unexplored polynya-formation mechanism: an Ekman transport of salt across a jet girdling the northern flank of the Maud Rise. Such transport was driven by intensified eastward surface stresses during 2015–2018. Our results illustrate how highly localized interactions between wind, ocean flow and topography can trigger polynya formation in the open Southern Ocean.

## Teaser

Local-scale Ekman dynamics enabled the formation of a rare open-ocean polynya within the Antarctic sea ice field of 2016 and 2017.

## INTRODUCTION

Polynyas are openings in the sea ice during winter that expose relatively warm ocean waters to a much colder atmosphere, resulting in large heat fluxes that cool the water column and can sustain deep vertical convection that ventilates the ocean interior (*1*, *2*). Coastal polynyas along the Antarctic margins are regular occurrences and are formed by strong katabatic winds that push sea ice away from the coast (*3*). In contrast, open-ocean polynyas in the Southern Ocean are rare events, often associated with surface salinity anomalies that initiate deep convection (*4*). Such convection connects the mixed layer with the warmer circumpolar deep water (CDW), which is found at relatively shallow depths of 200 to 500 m within the gyres of the subpolar Southern Ocean (*5*). In the subpolar Southern Ocean, salinity predominantly governs stratification (*6*). When surface stratification is eroded by positive salinity anomalies, induced by a range of both large- and local-scale factors, deep convection initiates. The extent of this convection is then determined by thermobaric and cabbeling effects (*7*, *8*).

The 1970s saw a large and persistent polynya in the Weddell Sea, termed the Great Weddell Polynya, which had its origins in the Maud Rise region (*9*). The Maud Rise is a subsurface seamount, the shallow bathymetry of which traps a column of cooler and denser waters, forming a Taylor cap surrounded by warm deep water (WDW) (*10*), which is derived from the CDW flowing via the Weddell Gyre into the Maud Rise region (*11*). This inflow, upon interacting with the topography, generates a halo of warm and saline WDW around the seamount (*12*, *13*) associated with a particularly weak stratification. This reduced stratification makes local conditions favorable for the formation of open-ocean polynyas (*14*), referred to as Maud Rise polynyas. The emergence of a Maud Rise polynya may be necessary to form a larger Weddell polynya, as surface salinity anomalies from the Maud Rise region are carried downstream into the central Weddell Sea to trigger convection there (*15*). The formation of such open-ocean polynyas affects the regional carbon and heat budgets (*16*, *17*). Density anomalies, exported from the Weddell polynya, could influence the stratification of remote regions of the global ocean (*18*).

**Fig. 1.**
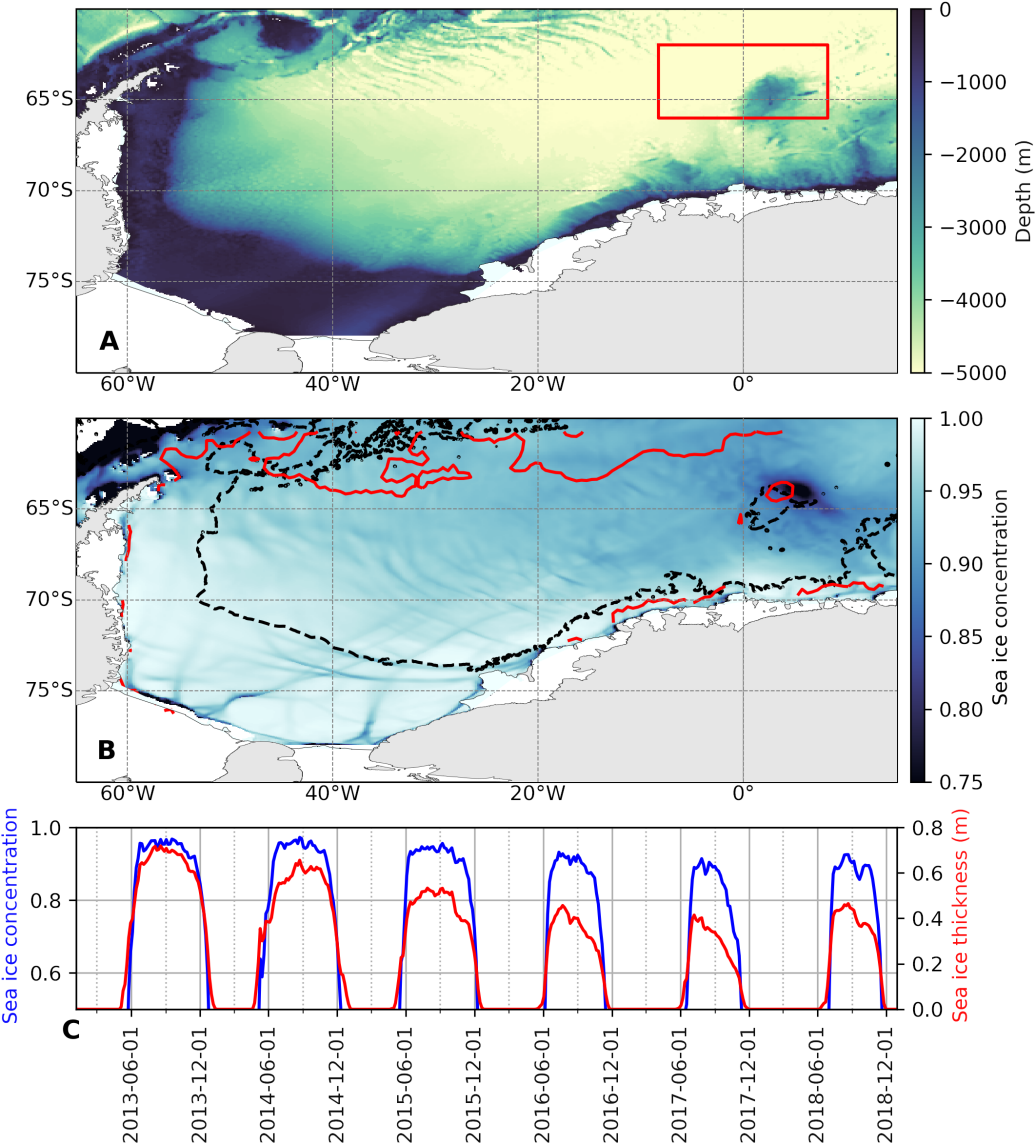
Maud Rise within the Weddell Sea. (**A**) Bathymetry of the Weddell Sea as represented in SOSE. The Maud Rise is enclosed within the red box (study region). (**B**) Modeled sea ice concentration (SIC) averaged over September2017 from SOSE output, showing the formation of the Maud Rise polynya. The red contour is the September-averaged SIC from satellite observations, plotted for a SIC of 0.75. The black contours indicate the 3000-m isobath. (**C**) Area-averaged SIC [over the area enclosed by the red lines in (A)] and effective sea ice thickness (sea ice volume per unit area) from SOSE.

Deep convection in the open Southern Ocean, and in the Weddell Sea in particular, is thought to have been more common in the pre-industrial era (*19*). The intensification and poleward shift of the Southern Hemisphere westerlies due to anthropogenic climate forcing have resulted in the persistence of a positive phase of the southern annular mode (SAM), which is associated with a warmer and wetter atmosphere, fresher surface ocean, and stronger, convection-inhibiting vertical stratification (*19*, *20*). Nevertheless, the winter of 2016 saw a polynya opening late in the season, and a relatively larger and more persistent polynya emerged during the winter of 2017 (*21*). Remotely sensed sea ice concentration from the National Snow and Ice Data Center (*22*) and Southern Ocean state estimate (SOSE)–generated (see Materials and Methods) sea ice fields show that the polynya initially formed over the northern flank of the Maud Rise in 2017 (Fig. 1B). Both the 2016 and 2017 polynyas remained broadly restricted to the Maud Rise region, with neither becoming as large as the Weddell polynya of the 1970s.

Francis *et al.* (*23*) showed that the 2017 Maud Rise polynya, hereafter referred to as the MRP17, was associated with exceptionally strong storms that induced a divergence of sea ice. This mechanism could explain the immediate trigger for the polynya, but it does not explain how the water column stratification became sufficiently weak to support deep convection and an enduring polynya. The long-term positive SAM trend has also been linked to the flow of anomalously warm and moist air, termed atmospheric rivers, into the Southern Ocean (*24*). Francis *et al.* (*25*) hypothesized that such atmospheric rivers caused sea ice thinning during the MRP17 event, by enhancing downward energy flux in the Maud Rise region. However, the melting of sea ice would have enhanced upper-ocean stratification and inhibited deep mixing, so it cannot explain the deep mixing that hydrographic observations reveal to have occurred in association with the polynya event (*26*).

Another possible polynya trigger was highlighted by Campbell *et al.* (*26*), who proposed that the anomalously high cyclonic wind-stress curl seen in the Weddell Sea during 2015–2016 may have enhanced vertical transport of salt into the mixed layer, eventually destabilizing it and setting up deep convection. The anomalous wind-stress curl corresponded with a prolonged period of positive tendency in the SAM (*27*), leading to a freshening of the Southern Ocean’s surface. This freshening effect was linked to the relatively limited extent of the MRP17 event compared to the events in the 1970s and the absence of a polynya reopening in 2018 (*28*).

Maud Rise polynyas are preceded, up to 4 months in advance, by an anomalous sea ice thinning early in winter that is concurrent with enhanced cyclonic winds, reduced sea surface heights, and a stronger Weddell Gyre circulation (*29*). High-resolution models indicate that remote density anomalies can be advected onto the Maud Rise, affecting the stratification over the region (*13*, *30*). Because positive salinity anomalies were associated with all simulated Maud Rise polynyas, local destratification via the remote advection of such anomalies was put forward as a possible mechanism for polynya initiation. Analysis of sparse observations available around Maud Rise confirms the presence of a large interannual variability in local circulation and stratification, in particular along the northern flank of Maud Rise (*13*).

In summary, previous studies have explored how the large-scale atmospheric and oceanic circulations in the Weddell Sea region may have triggered and sustained the MRP17 event, but a complete picture is still missing. Local-scale processes across the flanks of the Maud Rise can exert a substantial influence on local stratification, due to the flow’s interaction with topography. The isolation of the Taylor cap, and the doming isopycnals that connect the cap with the warmer waters surrounding Maud Rise, can help initiate convection (*31*, *32*). Further, the formation of cyclonic eddies along the Rise’s flanks can impose a divergent stress upon sea ice conducive to polynya formation (*12*, *33*). Here, we assess the role of these local-scale processes in triggering and sustaining the Maud Rise polynya. We show that a hitherto overlooked mechanism, a frictionally forced cross-frontal Ekman transport of salt, was an essential factor in initiating the MRP17 event over the northern flank of the Maud Rise.

To do this, we examine the drivers of the MRP17 event in the 1/6 SOSE (see Materials and Methods). We apply a potential vorticity (PV) framework to quantify the effects of surface buoyancy fluxes and frictional stresses on upper-ocean stratification. We show that the weakening of stratification leading to polynya emergence can be traced back to conditions up to 2 years before the MRP17 event and that destratification was driven by a combination of vertical mixing of salt and a cross-frontal Ekman transport forced by surface frictional stresses. These two processes led to an increase in salinity and buoyancy loss in the mixed layer from June 2015 to 2018, priming the region for the formation of a persistent polynya.

## RESULTS

### Upper-ocean salinity balance

**Fig. 2.**
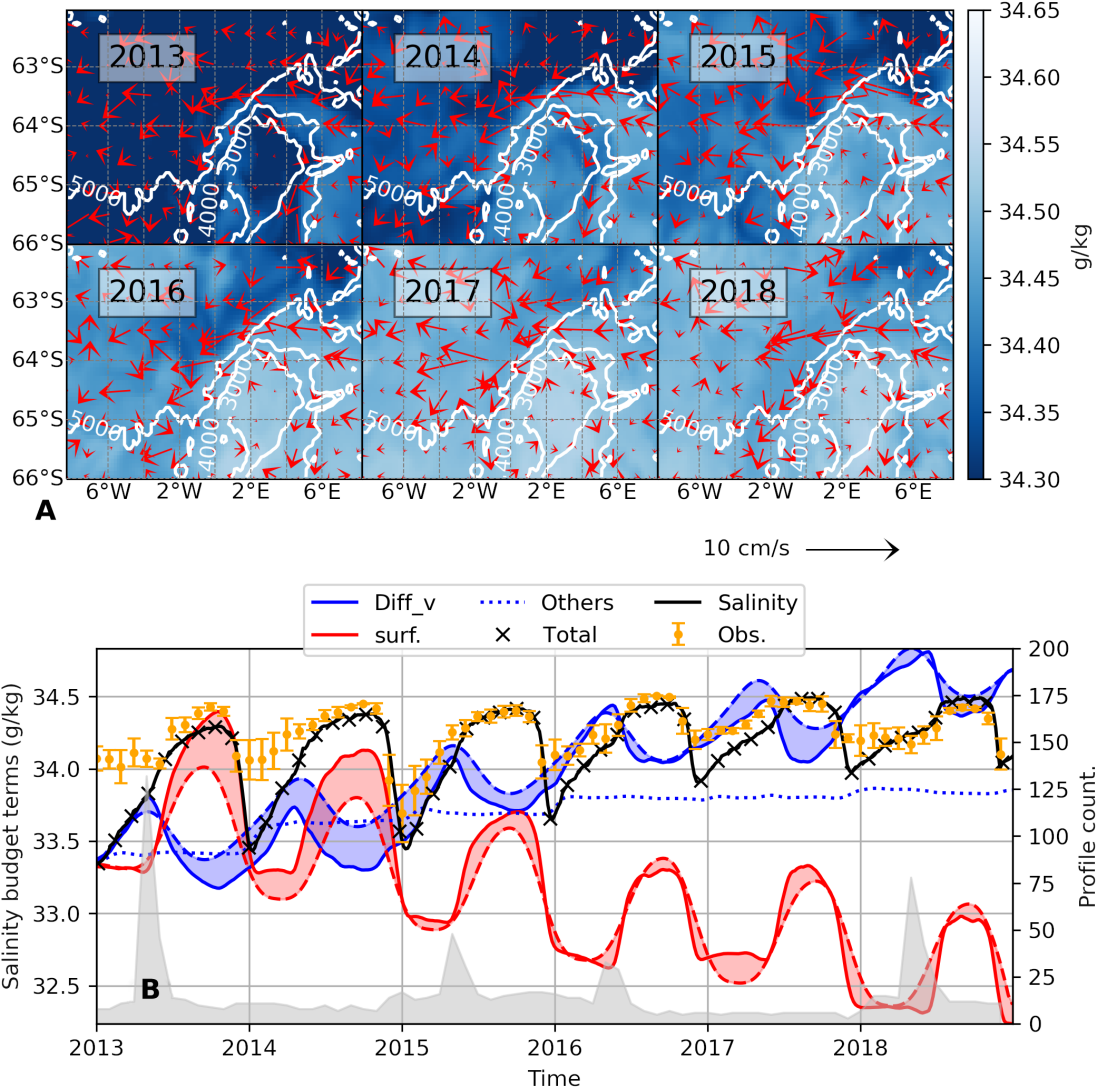
Surface salinity from the SOSE. (**A**) September-mean surface salinity, computed as upper 20 m averages, for the years 2013–2018. Vectors are the September-mean geostrophic velocity. White contours show isobaths. (**B**) Salinity balance, spatially averaged [over the area in (A)] and cumulatively summed in time, and vertically averaged in the uppermost 20 m. The black curve is the salinity, the “x” markers denote the sum of the salinity balance terms, and the orange markers are the monthly mean of observed salinities (spatially averaged, with their associated monthly SD). The salinity balance terms for vertical diffusion and surface fluxes are shown, while the other terms are summed up and displayed as “others” (see Materials and Methods). Continuous lines are the instantaneous values, while the broken lines are the monthly means. The shading emphasizes the difference between these two lines where shading above dashed lines indicates a positive anomaly. The light gray shading at the bottom is the number of observed profiles plotted against the right-hand *y* axis.

Campbell *et al.* (*26*) showed that the Weddell Sea experienced an enhanced vertical transport of salt into the mixed layer in 2015–2016, which they attributed to anomalously high cyclonic wind-stress curl. This increase in mixed-layer salinity led to a reduction in stratification, preconditioning vertical convection and enabling the formation of a polynya. SOSE reproduces this increase in near-surface salinity (computed using vertical averages of the uppermost 20 m of the water column) and indicates its progressive occurrence between 2013 and 2018 (Fig. 2A). The displayed salinities are time averaged in the month of September, which is the month with the greatest sea ice extent in the Southern Ocean and, consequently, the month with the highest surface salinity and weakest stratification. Vectors show the geostrophic velocity field (computed using vertical averages) temporally averaged over each September. The westward-flowing southern limb of the Weddell Gyre is readily apparent on the northern flank of the Maud Rise, as is an eastward-flowing retroflection along the 3000-m isobath. This northern-flank area and its associated jet will be the main focus of our study.

The salinity balance term primarily responsible for the increase in surface salinity over our study region is the vertical diffusion term, which accounts for the vertical mixing of salinity (Fig. 2B, blue curve). The seasonality of the surface salinity is dominated by the surface fluxes (Fig. 2B, red curve). Surface fluxes tend to reduce salinity during our study period, as sea ice is imported to the region from coastal polynyas and subsequently melts each summer (*34*, *35*). Vertical mixing (“Diff_v,” blue curve) counteracts the surface flux and injects salinity into the surface layer across the model-run period. This is consistent with the injection of salinity via entrainment of salt from the underlying WDW, as seen in fig. S6. During the times when the 27.74 kg m^−3^ isopycnal (used here as a proxy for the WDW layer) shoals close to the mixed layer, we see a drop in the temperature and salinity of the isopycnal, indicating that heat and salt from the WDW layer were entrained into the mixed layer. The WDW layer shoals until it is as shallow as the winter mixed layer depth (defined in the Materials and Methods) during 2016, 2017, and 2018. This is the period with the lowest WDW temperatures and salinities and with the highest surface salinities, again indicative of turbulent entrainment of heat and salt from WDW into the mixed layer (especially marked during the winters of 2016, 2017, and 2018; see fig. S6).

It is noteworthy that there is a progressive increase of near-surface salinity over the Taylor cap (corresponding to topography shallower than 3000 m) compared to the northern flank of the seamount (more clearly seen in fig. S11). During 2013–2015, the Taylor cap exhibited lower near-surface salinity than the northern flank; yet, from 2016 to 2018, salinity over the Taylor cap exceeded that of the northern flank. Cross-seamount transects centered at 3°E (presented in fig. S11) illustrate the near-surface salinity buildup over the seamount, reaching depths of approximately 100 m. The implications of this accumulation of salt in the upper ocean above the seamount for polynya formation are explored in subsequent sections.

### Spatial configuration of the wintertime salinity balance

**Fig. 3.**
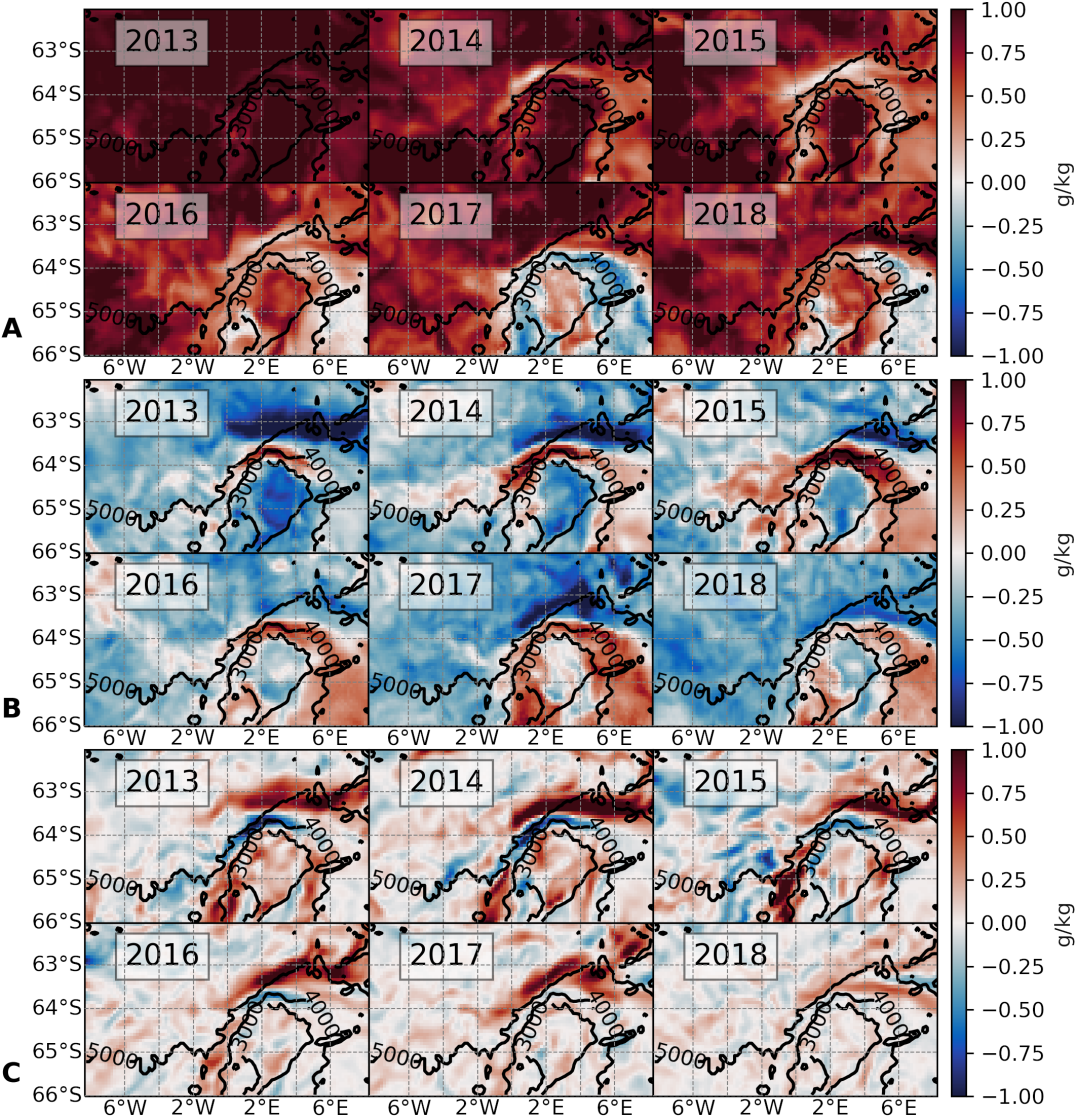
Upper ocean salinity balance. (**A**) Surface salinity flux, time integrated from March to September for years 2013–2018 (∫*G*_surf_*dt*; see Materials and Methods). (**B**) Vertical diffusion of salinity, vertically averaged over the uppermost 20 m, and time integrated from March to September ( $\frac{1}{20}\int_{z=-20}^{z=0}\int \text{Diff\_}v\text{dtdz}$ ; see Materials and Methods). (**C**) Horizontal Ekman transport of salinity, vertically averaged over the Ekman layer (average of 22 m) and time integrated from March to September (∫Ek_adv_*dt*; see Materials and Methods). Positive values indicate increase in surface salinity.

The upper-ocean salinity balance described above can be synthesized with spatial maps of selected key balance terms, temporally integrated over the sea ice growth period of March to September (Fig. 3). Surface waters increase their salinity during this period as a result of the wintertime sea ice growth (i.e., brine rejection; Fig. 3A), with the salinity increase then being redistributed into the rest of the mixed layer via the vertical diffusion term (Fig. 3B). These surface fluxes are generally weaker over the “halo” region of the Maud Rise, as seen in Fig. 3A; and the surface salinity fluxes are actually negative between 2016 and 2018, indicating early sea ice melting in those years. The halo has been previously characterized by de Steur *et al.* (*12*) as a region of warm water surrounding the cooler Taylor cap over the Maud Rise, and forms as the westward-flowing southern limb of the Weddell Gyre impinges upon the Rise while conveying relatively warm and saline WDW. The near-surface layer in the halo region receives an input of salinity by vertical mixing (Fig. 3B), consistent with local entrainment of this saline variety of WDW into the mixed layer (black line in Fig. 2C).

The horizontal Ekman transport of salinity provides another contribution to the upper-ocean salinity balance (Fig. 3C). Over the Taylor cap, Ekman advection leads to a buildup of surface salinity in the years leading up to the polynya. Over the northern flank of Maud Rise, in 2013–2014, we find that the horizontal Ekman transport is negative around the 4000-m isobath, i.e., it reduces near-surface salinity and strengthens stratification there. In contrast, in 2016–2018, the Ekman-induced loss of salinity over the 4000-m isobath weakens and eventually vanishes, whereas the band of Ekman-induced salinity gain that had previously been to the north of the 4000-m isobath moves poleward, closer to the Taylor cap. This signals a role of surface frictional processes in destratifying upper-ocean waters immediately preceding and during the MRP17 event and prompts a more rigorous examination of the dynamical controls of local stratification.

### PV as a proxy for stratification

**Fig. 4.**
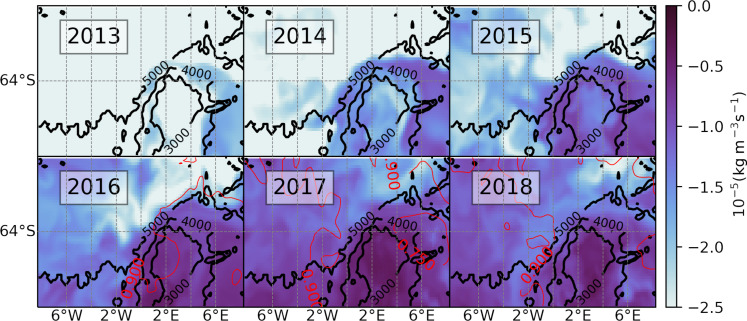
Wintertime PV. PV, vertically integrated over depths of 0 to 500 m, and averaged over the month of September for years 2013–2018. The red contours denote SIC averaged over the month of September, plotted at intervals of 0.1.

We next examine the Ertel PV (see Materials and Methods), which is used here as a conserved and dynamically informative proxy for stratification. PV is generally negative in the Southern Hemisphere, and values closer to zero indicate reduced stratification. Budgets of PV provide insight into the causes of the upper-ocean stratification decrease that underpinned the polynya’s formation. Near-surface PV averaged over the month of September during years 2013–2015 (Fig. 4) is commonly weaker (i.e., lower in magnitude) over the halo surrounding the Maud Rise. This is consistent with a weakening of upper-ocean stratification in the halo via enhanced salinity entrainment (Fig. 3B), following interaction of the Weddell Gyre with the Rise (*12*, *33*).

In 2013–2015, upper-ocean PV and stratification progressively weaken (i.e., approach zero) over the Taylor cap and the halo, reaching minimum magnitudes in 2016–2018. It is in this latter period that SIC reduces over both the Taylor cap and the halo (Fig. 1C), as expected from the weaker stratification promoting entrainment of heat from the underlying WDW into the mixed layer.

**Fig. 5.**
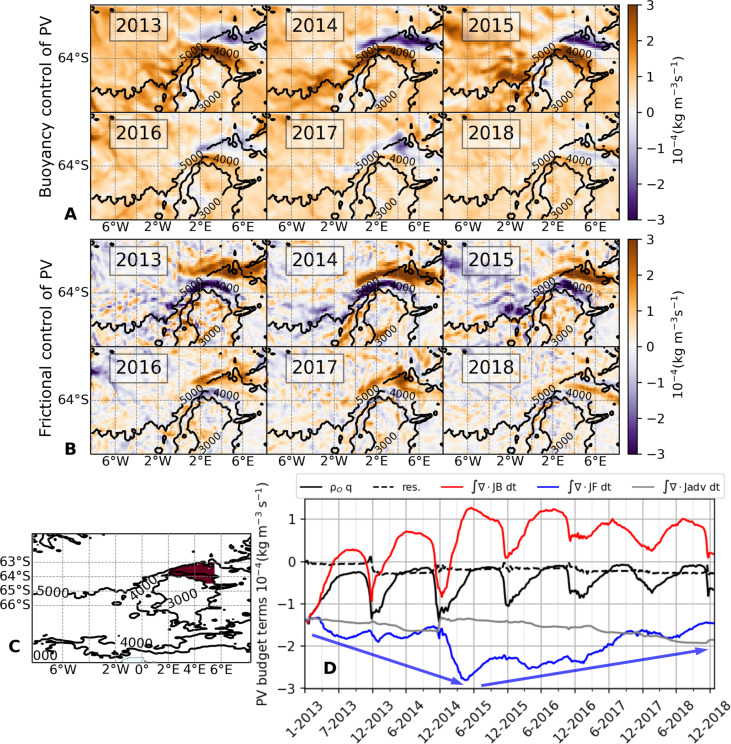
Buoyancy and frictional control of stratification. (**A**) −∫ ∇ ⋅ *J*^B^*dt*: the PV change induced by buoyancy forcing, time integrated over the period from March to September for each year between 2013 and 2018. (**B**) −∫ ∇ ⋅ *J*^F^*dt*: the PV change due to frictional processes, time integrated over the period from March to September for each year between 2013 and 2018. (**C**) Selected area over the northern flank of Maud Rise over which PV budget terms are spatially averaged. (**D**) Components of the PV budget spatially averaged over area in (C). The black curve is PV, the red curve is the PV change due to buoyancy forcing (*J*^B^); the blue curve is the PV change due to frictional processes (*J*^F^, equivalent to a frictionally forced cross-frontal transport of PV), the gray curve is the three-dimensional advection of PV (J^A^), and the broken black line is the PV budget residual. The blue arrows visually approximate the slope of the *J*^F^ curve and indicate that this slope changes sign in mid-2015.

To elicit the drivers of these near-surface PV and stratification changes, we decompose the PV evolution into its component terms associated with buoyancy forcing (*J*^B^), frictional forcing (*J*^F^), and advection of PV (*J*^A^), as detailed in Materials and Methods (“PV framework” section). The buoyancy term (Fig. 5A), time integrated from March to September, is a measure of the rate at which buoyancy forcing acts to destratify (positive values) the upper water column. Throughout the Maud Rise area, *J*^B^ is indicative of destratification, which conforms with the expected stratification impact of brine rejection during sea ice formation in winter.

An exception to this general pattern occurs over the 5000-m isobath on the Rise’s northern flank, where *J*^B^ contributes to restratification. While *J*^B^ is typically dominated by surface buoyancy fluxes (*36*), regions of negative *J*^B^ in our study do not align with such negative surface salinity fluxes (Fig. 3A). Since substantial vertical mixing between near-surface waters and the underlying WDW has been demonstrated for the halo (Fig. 3B), it is thus likely that this mixing is responsible for the observed near-surface buoyancy reduction in that area.

This interpretation is substantiated by directly comparing regions of negative *J*^B^ with those where the sum of the surface salinity flux and vertical diffusion of salinity is negative (fig. S7). As the upper ocean becomes saltier from sea ice formation, it begins to entrain heat and salt from the WDW below. This entrained heat provides a negative feedback on destratification by melting sea ice, a mechanism known as the “thermal barrier” effect, which prevents destabilization (*37*–*40*). From 2016 to 2018, the area of buoyancy gain shifted poleward toward the Taylor cap (Fig. 5A and fig. S7), suggesting that the interplay between the surface salinity flux and vertical diffusion of salinity restratified the upper ocean on the Rise’s northern flank.

If *J*^B^ indicates buoyancy gain and upper-ocean reinforcement of PV and stratification over the northern flank during 2017, then what drove the weakening of PV and destratification that occurred at that time? The answer lies with the other key factor controlling PV: the frictional term (*J*^F^; Fig. 5B). *J*^F^, time integrated over the period from March to September, is largely representative of the cross-frontal Ekman transport of buoyancy (*36*). The greatest magnitudes of *J*^F^ are apparent in locations where the strongest fronts in the region are found, that is, over the northern flank of the Maud Rise.

From 2013 to 2015, *J*^F^ enhanced PV and stratification over the 4000-m isobath north of the Maud Rise, while reducing PV and stratification over the 5000-m isobath (Fig. 5B), typically countering *J*^B^. Over 2016–2018, *J*^F^ diminished substantially over the 4000-m isobath north of the Maud Rise, and the zone of positive, destratifying *J*^F^ moved poleward toward the 3000-m isobath. This shift corresponds with the poleward translation of the positive Ekman contribution to the upper-ocean salinity balance revealed by Fig. 3C, with *J*^F^ (Fig. 5B) and Ekman salinity transport (Fig. 3C) exhibiting similar spatial patterns. This suggests that a poleward (toward the Taylor cap) repositioning of the front on the northern flank of the Rise occurred after 2016.

The PV budget components over the Rise’s northern flank are spatially averaged in Fig. 5D. We note here that the budget does not close perfectly and that budget residuals are relatively larger during the month of December in most years (broken line, Fig. 5D). We attribute this to the budget’s offline computation using 5-day averaged fields, which miss some of the higher-frequency changes occurring during the sea ice melt period. At any rate, the residual is small relative to other budget terms and remains constant through the rest of the year. This gives us confidence that the budget is useful for the purpose of diagnosing the factors contributing to destratification of the water column during the MRP17 event.

The annual cycle in near-surface PV, marked by seasonal buoyancy loss in the upper ocean, is delineated by the *J*^B^ term (red curve; Fig. 5D). In addition, a trend toward weaker PV over 2013–2015 is apparent, predominantly associated with the *J*^B^ term. This aligns with the broad intensification of the Weddell Gyre, which facilitated injection of salt into the mixed layer (as previously indicated in Fig. 3B) and a corresponding decrease in near-surface stratification during 2013–2015. In 2017, *J*^B^ exerted a minimal destratifying effect and commenced restratifying earlier in the year (July 2017), suggesting that vertical mixing and surface fluxes alone do not account for the subsequent destratification linked to the MRP17 occurrence.

Instead, such destratification was caused by the *J*^F^ term, which reverted from a negative tendency over 2013–2014 to a positive tendency thereafter, beginning in May 2015 (blue line, Fig. 5D). This indicates that during the winters of 2016–2018, the cross-frontal Ekman transport of buoyancy more than compensated for the buoyancy gain due to early sea ice melting and was thus an essential factor for the reduction of upper-ocean PV and stratification over the northern flank of the Maud Rise.

### Surface stresses and density gradients

**Fig. 6.**
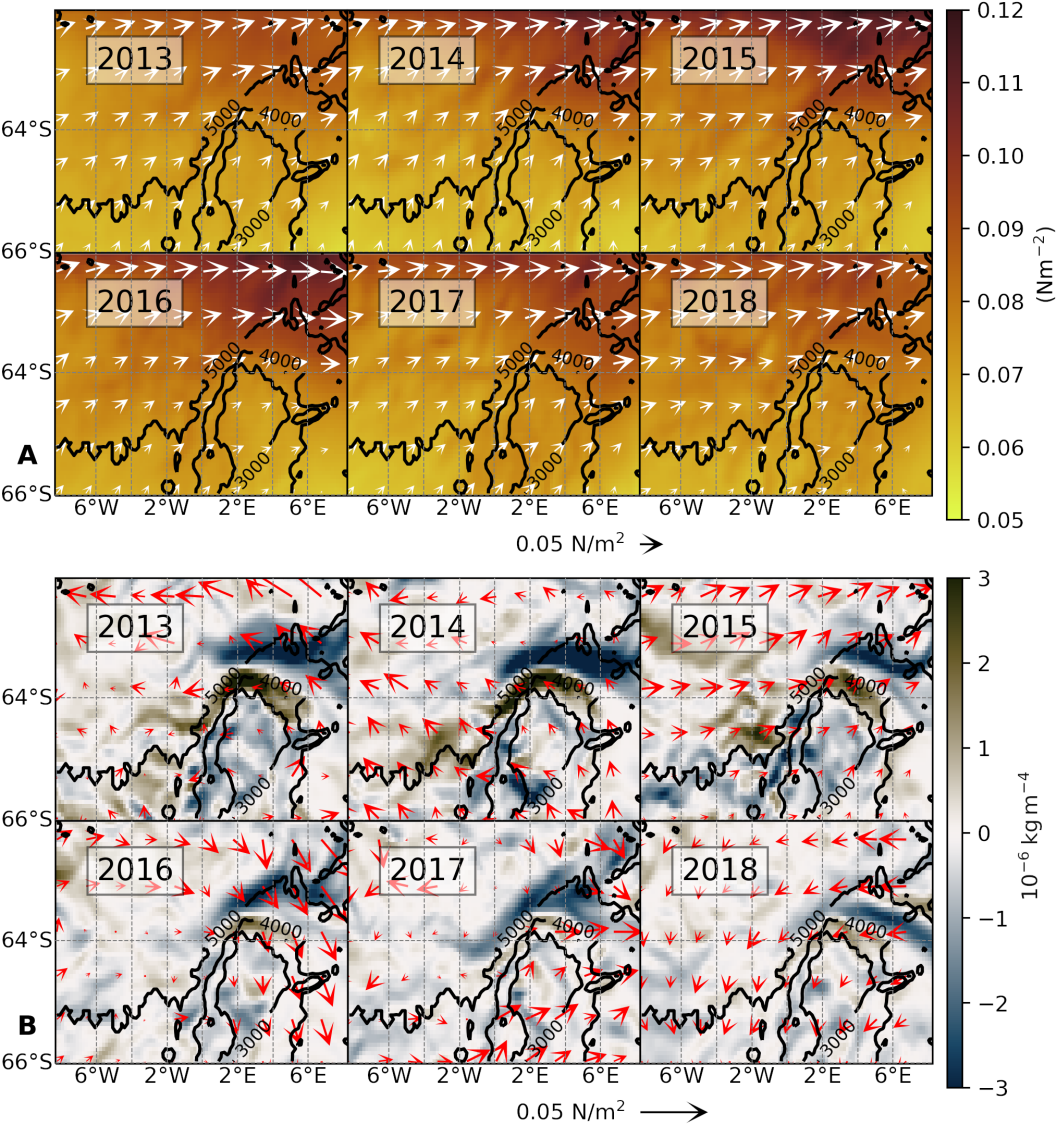
Surface friction and density gradient. (**A**) Net ocean surface stress (from SOSE; inclusive of sea ice effects), time averaged over the period from March to September for the years indicated. The shading represents the magnitude and the arrows are the associated vector field. (**B**) Modeled meridional gradient of surface density, time averaged from March to September. The vectors show the anomalies in net surface stress (time averaged from March to September; relative to the 6-year mean).

The destratifying Ekman transport highlighted by our previous analyses depends on two factors: (i) the net ocean surface stress forcing the Ekman velocity and (ii) the horizontal gradient of density. To identify the extent to which each of these factors was responsible for the anomalous Ekman transport of 2015–2018, we examine the net surface stress field and the meridional gradient of density (Fig. 6). Over the northern flank of the Maud Rise, the stress field shifts southward (closer to the Taylor cap; Fig. 6A). In the same area and period, the surface stress becomes aligned more directly along the eastward direction (vectors in Fig. 6B), which is oriented along the frontal jet such that denser waters from the Taylor cap are advected northward over lighter waters (*41*), leading to a destabilizing Ekman effect over the upper water column consistent with our preceding diagnostics. Concurrently, the meridional gradient of surface density initially builds up across the northern flank of the seamount in the years 2015 through 2018 (poleward migration of blue area on the northern flank; Fig. 6B). This density gradient extends to depths of up to 200 m (as seen in meridional transects of salinity in fig. S11). Subsequently, the surface density gradient diminishes over 2018 (Fig. 6B), in agreement with an enhanced salinity transport across the front.

In summary, the destratifying Ekman transport leading to the MRP17 event was due to a combination of the buildup of salt over the Taylor cap and the eastward intensification of the stress field.

### Drivers of (de)stratification

**Fig. 7.**
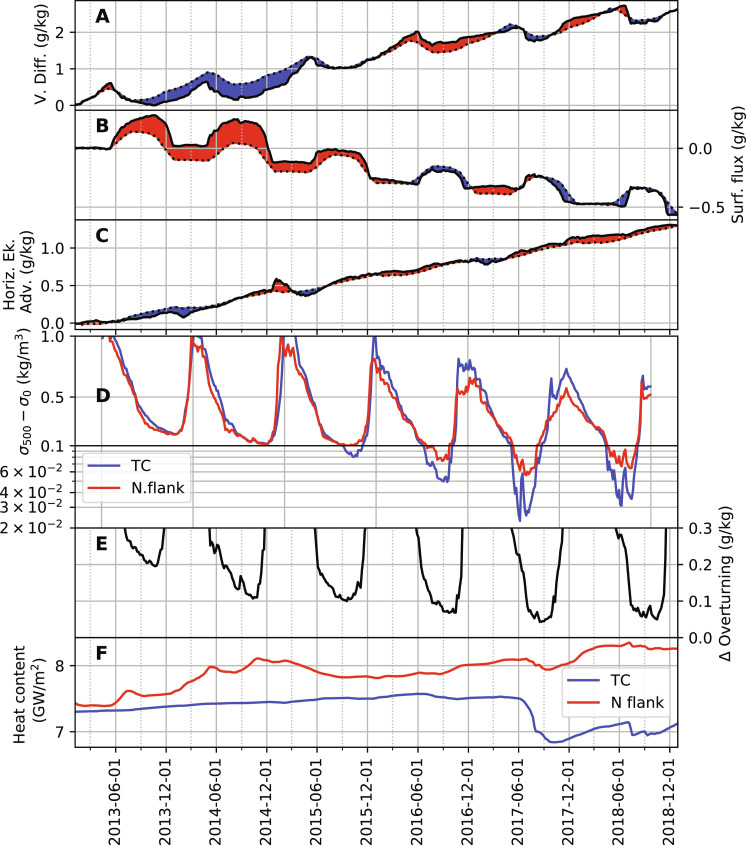
Spatially averaged properties of the northern flank. (**A**) Time-integrated vertical diffusion of salinity (upper 20 m average), (**B**) time-integrated surface flux of salinity, and (**C**) time-integrated horizontal Ekman advection of salinity. Red shades denote positive anomalies, and blue negative with respect to monthly climatological averages. The northern flank is as defined in Fig. 5C. (**D**) Difference between potential density at depths of 500 m and the surface, averaged over the Taylor cap (blue line) and the northern flank (red line). Note that the *y* axis switches to a logarithmic scale below values of 0.1. (**E**) Δ_ov_ (see Materials and Methods), defined as the difference between the overturning limit of salinity and the mixed-layer salinity. (**F**) Oceanic heat content over the Taylor cap (TC) and the northern flank of Maud Rise (N flank), integrated over depths of 200 to 1000 m.

To further elucidate what caused the upper-ocean destratification during the MRP17 event, and specifically over the northern flank of Maud Rise, we now inspect three important terms of the near-surface (uppermost 20 m) salinity balance (the vertical diffusion, the surface flux, and the horizontal Ekman advection; see Fig. 7). As discussed earlier, the vertical mixing of salinity acts to add salt to the near-surface layer, and the mixing is elevated during 2015–2018 (Fig. 7A). The surface flux acts to balance the vertical mixing (Fig. 7B) and becomes anomalously low during 2016–2018, consistent with the weaker sea ice at that time. The horizontal Ekman advection adds salt to the near-surface layer throughout 2013–2018, and is especially enhanced from June 2015 to 2018 (Fig. 7C). Although our use of 5-day averages means that the water column appears to never become fully unstable, the weakest stratification is found during the winters of 2016 through 2018, with the difference in potential density between the surface and 500 m reaching as little as 0.02 kg/m^3^ (Fig. 7D). Deep mixing of heat and salt takes place over the Taylor cap as a result (fig. S8). Thus, the leading-order drivers of upper-ocean salinification are the vertical mixing and horizontal Ekman advection of salinity.

We define Δ_ov_ as the difference between the overturning limit of salinity and the mixed-layer salinity (“salt deficit,” see Materials and Methods). Modest values of Δ_ov_ indicate that a small salinity increase would be sufficient to initiate overturning in the upper water column. Δ_ov_ is smallest in 2017 and 2018 (Fig. 7E). The very low values of Δ_ov_, which reaches minima of ~0.05 g/kg in September 2017/2018, are comparable to the anomalies in the horizontal Ekman advection (Fig. 7C) during those periods. The comparable size of these terms denotes that anomalies in the Ekman term are ample enough to contribute substantially to destratifying the upper ocean, so that the Ekman transport is an important player in eroding the stratification over the Rise’s northern flank.

The condition of diminished near-surface stratification extends into 2018, as reflected in the low Δ_ov_ values then. Although 2018 saw less sea ice cover, the decline in sea ice then was not as marked as in 2017, which recorded the lowest winter sea ice concentration for the region (Fig. 1C and fig. S8A). To assess the factors behind this sea ice reduction, we assess the subsurface oceanic heat content (Fig. 7F; integrated over depths of 200 to 1000 m) over the Rise’s northern flank and within the Taylor cap (i.e., the area shallower than 3000 m). During 2017, there was a slight decrease in heat content over the northern flank, compatible with polynya activity and the initiation of deep convection, which ventilates heat to the atmosphere. After December 2017, the heat content recovered on the northern flank, likely due to the Weddell Gyre’s delivery of WDW to the Maud Rise flanks offsetting the heat loss. Following its appearance on the flanks, the polynya extended over the Taylor cap, where heat content diminished amid the MRP17 event and remained low through 2018. This sustained reduction in heat content partly stemmed from the seamount’s topographic sheltering from the southern limb of the Weddell Gyre, which largely bypasses the Taylor cap. In addition, post-MRP17, the waters immediately beneath the mixed layer cooled by approximately 1°C (fig. S8D), thereby limiting heat input to the mixed layer in the following winter. Vertical mixing of salinity into the mixed layer in 2018 was also less intense compared to earlier years (Fig. 7A). We deduce that this combination of modest lateral heat resupply to the Taylor cap and curtailed salinity mixing into the mixed layer accounts for the absence of a re-formed polynya in 2018, despite the prevailing weak stratification during that time.

**Fig. 8.**
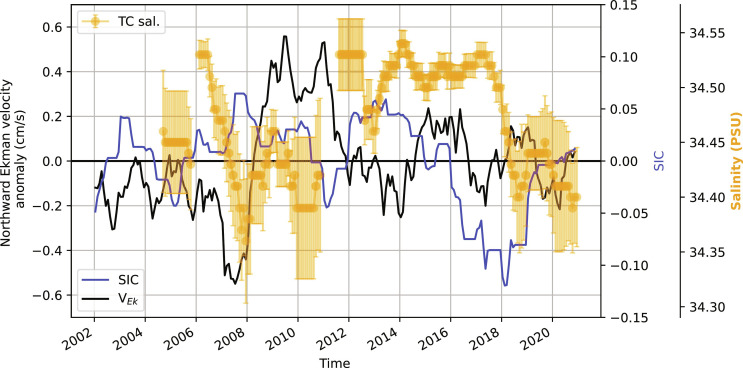
Synthesis of observations of the northern flank of Maud Rise. Seasonal anomaly in the northward Ekman velocity (black curve; derived from remotely observed ocean surface stresses; see Materials and Methods), sea ice concentrations (SIC; blue curve; seasonal anomaly), and monthly mean surface salinity observed by profiling floats and tagged seals over the Taylor cap (orange markers; error bar indicates ±1 SD; see fig. S5 for profile locations).

To provide independent supporting evidence for our findings and contextualize them over a longer period, we analyzed remotely observed sea ice concentration (*22*) and velocity (*42*), winds from the ERA-5 dataset (*43*), and surface salinity observations gathered by profiling floats over the Taylor cap (profile locations in fig. S5) during the 2002–2020 period (Fig. 8). While Ekman velocity anomalies were northward during 2008–2012 and 2014–2018, a northward transport of salt from the Taylor cap onto the northern flank can only occur when salinity is elevated over the cap. Such high salinity was observed only during the period from 2014 to 2017, consistent with SOSE model diagnostics. Short-lived elevations in Taylor cap salinity were also recorded at the beginning of 2006 and the close of 2012. These episodes were transient, lasting only a few months, in contrast to the extended duration of the high Taylor cap salinity event from 2014 to 2018.

## DISCUSSION

We have used a suite of direct observational products and an ocean reanalysis model that assimilates observations to investigate the processes leading to the 2017 Maud Rise Polynya’s formation. It is important to note that SOSE, with a resolution of 1/6° × cos(lat), has approximately 8 km by 8 km grid cells over the Maud Rise, a scale that only partially resolves mesoscale eddies in the area (see Materials and Methods for more details). While eddies are a possible mechanism for cross-frontal mixing not entirely captured in this study, SOSE successfully represents other key dynamics within the region. The model reproduces the westward-flowing jet on the Rise’s northern flank and the eastward-flowing retroflection adjacent to the seamount. In addition, the model depicts the accumulation of salt over the seamount, a finding supported by in situ float data. The model also captures the larger Weddell Gyre’s spin-up and the increased cyclonicity over the Maud Rise area. The anomalous wind patterns that facilitate enhanced northward Ekman velocities are imposed using ERA-5 reanalysis, with only minor modifications resulting from the data assimilation process. Last, SOSE captures the initial triggering of polynya formation over the northern flank.

**Fig. 9.**
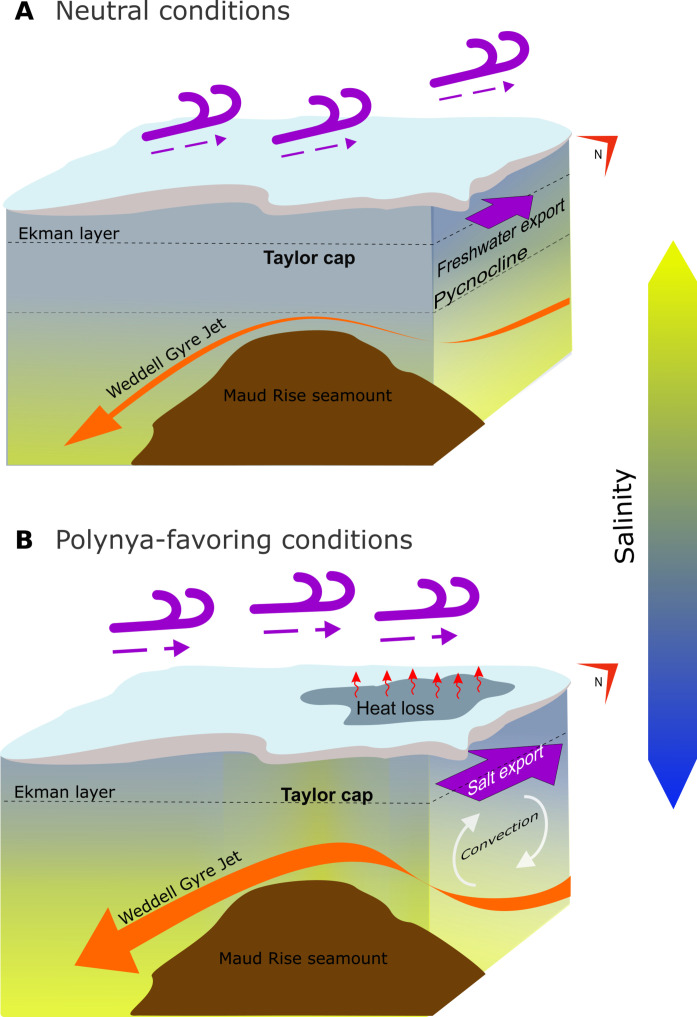
Schematic of the key processes detailed in this analysis, leading to the formation of the Maud Rise Polynya in 2017. Note that the vertical scale of the upper Ekman layer is exaggerated for clarity. (**A**) Depicts the years 2013 to mid-2015, characterized by a fresher surface over the seamount, weaker eastward stress, and Ekman transport of freshwater from the seamount to the northern flank. (**B**) Represents the years mid-2015 to 2018, featuring a saline upper ocean over the seamount, an intensified Weddell Gyre jet, stronger eastward surface stress, and Ekman transport of saline waters onto the northern flank, facilitating convection in that area during the polynyas of 2016 and 2017. Credit: Claudia Ofelio.

The sequence of processes leading to Maud Rise Polynya emergence in 2016–2017 is synthesized in Fig. 9. In the years 2013–2015, the Taylor cap accumulated salt near the surface due to enhanced vertical mixing and enhanced eddy advection of salt anomalies from the flanks to the seamount, enabled by a stronger jet due to the spin-up of the Weddell Gyre (*12*, *13*). During polynya formation in 2017 over the Rise’s northern flank, there was a net buoyancy gain due to sea ice melt that compensated for the vertical mixing of salinity. From mid-2015 to 2018, surface stresses intensified eastward to transport salinity from the Taylor cap onto the northern flank, providing an additional source of near-surface salt that resulted in destratification and polynya formation in 2016 and 2017. Although the water column remained weakly stratified in 2018, a polynya did not re-emerge, possibly due to a reduction in heat content over the Taylor cap.

The Weddell Gyre’s intensification coincided with stronger cyclonic wind patterns over the Weddell Sea, especially above Maud Rise (*44*). These winds are associated with changes in the mean sea level pressure over the Southern Ocean (*23*). Sea ice is sensitive to variations in mean sea level pressure, which itself shows interannual variability influenced by the SAM (*45*). The intensification of these large-scale winds also accounted for the increased eastward stresses over the northern flank of Maud Rise (*26*), resulting in stronger northward Ekman velocities. Furthermore, the gyre’s acceleration played a key role in the buildup of heat and salt over the Taylor cap. This process was driven by the heightened eddy energy in the area, enhancing the transfer of heat and salinity anomalies onto the seamount (*13*). Hence, it is inferred that large-scale dynamics are intricately linked to the localized mechanisms that instigated the polynya’s formation, demonstrating a coordinated interplay between these scales in the polynya’s development.

The northern flank is especially favorable to the formation of polynyas, due to the flow’s interaction with topography enhancing vertical mixing of heat and salt. The dynamically isolated Taylor cap supports the buildup of salt anomalies, which can bring about elevated horizontal buoyancy gradients and thereby enable finescale processes to shape the stratification over the Rise and its flanks.

Crucially, during the years with reduced sea ice cover (2016–2018), the early melting more than compensated for the upward mixing of salinity and contributed to a net buoyancy gain over the northern flank of the Maud Rise. Hence, an additional source of salinity was necessary to destabilize the upper ocean. We identify this additional factor to be the cross-frontal Ekman transport of salt, which forced destratification of the upper water column, in particular over the Rise’s northern flank. The salt that built up over the Taylor cap from 2013 to 2015 was transported to the northern flank by eastward-intensified stresses from mid-2015 to 2018. This Ekman mechanism was superimposed upon the background large-scale effect of the gyre spinning up over the wider Weddell Sea region. Preconditioning by the cross-frontal transport provided the additional kick that was needed to initiate the polynya over the Rise’s northern flank. Once the polynya formed in 2017, it spread over the Maud Rise seamount, which was weakly stratified in 2016–2018. The cross-frontal transport anomalies began up to 2 years before the polynya opened in 2017.

Our analysis is consistent with the published literature identifying a large-scale spin up of the Weddell Gyre as a major factor in weakening upper-ocean stratification in the years leading up to the MRP17 event (*28*). Between 2013 and 2018, the Weddell Sea experienced a negative wind-stress curl anomaly that promoted shoaling of WDW and facilitated entrainment of salinity into the mixed layer. This resulted in elevated near-surface salinities and anomalously weak near-surface stratification (*28*). SOSE captures such an anomalously intense vertical mixing of salinity into the mixed layer, in line with the expected upward entrainment of saline WDW. This, in combination with horizontal Ekman advection of salinity, led to a buildup of salt in the upper ocean over the Taylor cap in the years leading up to the polynya. The impact of the Weddell Gyre’s large seasonal and interannual variability (*46*) on the hydrography and stratification of the Maud Rise merits further study.

Polar cyclones have been cited as a possible source of kinetic energy that helped entrain salt from the underlying WDW layer (*23*, *26*, *39*), and this entrainment would have been enhanced when WDW was already at an anomalously shallow depth (due to the spin up of the Weddell Gyre). However, salt entrainment is largely compensated through enhanced sea ice melting, likely due to the “thermal barrier” effect (*37*). It is possible that the surface stress anomalies that drove the anomalous cross-frontal Ekman transport could have been enhanced by the polar cyclones passing through the Maud Rise region. Our analysis cannot verify this, as SOSE archives 5-day averaged fields that filter out the higher frequencies associated with storms from the budget terms. In any case, since the effects of the storms accumulate in time (as there is no restoring negative flux of salinity after the passage of each storm), our analysis does include the cumulative impacts of these high-frequency terms to the extent captured by the ERA-5 winds used to force the model. Note that the critical factors in our analysis are the buildup of a salinity gradient across the Rise’s northern flank and the change in location and orientation of the mean wind stress in 2017, which could be driven by an increased storm activity.

Kurtakoti *et al.* (*30*) discussed another physically plausible mechanism for polynya formation, involving the transport of salinity anomalies from the Astrid Ridge to the Maud Rise and subsequent near-surface destabilization. SOSE does not show evidence for such a mechanism during MRP17. Thermobaricity may also play an important role in overcoming the salt deficit (*47*), and it is expected to be more important once a weak pycnocline has been established. We have described a process of preconditioning that is associated with the anomalous wind stresses over the northern flank of the Maud Rise that began almost 2 years ahead of the MRP17 event. Longer-term preconditioning may take place on time scales of decades too, as the subsurface heat reservoir builds up between polynya events (*48*). Future occurrences of polynya openings should also be affected by long-term trends in surface salinity forcing (*19*).

Our study brings to the fore the role of highly localized interactions between wind forcing, flow, and topography in shaping the upper-ocean stratification over the Maud Rise, and priming the area for formation of open-ocean polynyas. Specifically, frictionally driven cross-frontal salinity transport was a key factor in destabilizing upper-ocean stratification and forming the 2017 polynya.

## MATERIALS AND METHODS

### Southern Ocean state estimate

The ocean reanalysis model analyzed in this study is the SOSE, iteration 135, which is a run of the MITgcm model at a resolution of 1/6° for the period from 2013 to 2019, with an adjoint method to assimilate observations (*49*). The adjoint method iteratively solves for the least-squares fit of the oceanic general circulation with initial conditions and assimilated observations including CTD profiles from Argo floats and tagged seals (*50*), and satellite-based observations of sea level, surface temperature, and salinity (*51*). Atmospheric parameters (atmospheric fluxes of heat, freshwater, and momentum) from the ERA-5 solution (*52*) are prescribed at hourly intervals, using boundary layer bulk formulae as found in Large and Yeager (*53*). A sea ice thermodynamics model was implemented as found in Hibler (*54*). The adjustments applied to the winds via the adjoint method are smaller (fig. S1) than the known biases in ERA-5 over the Maud Rise (*55*). Further, SOSE is known to reduce biases in atmospheric reanalysis models (*56*). The ocean state estimate that is produced is internally consistent and conserves all the conservative physical quantities in the ocean. Moreover, SOSE is eddy permitting and capable of partly resolving mesoscale eddies. This capability has been validated through comparisons with satellite-observed sea surface height variations (*49*).

The Southern Ocean is poorly observed, making the validation of SOSE challenging. Although limited by spatiotemporal sampling biases, the best observational set of data available today are the objectively analyzed fields in the EN4.2 dataset, built from in situ CTD profiles from ships, floats, and tagged seals (*57*). These were used to validate the annually averaged surface salinities (vertically averaged in the upper 20 m) produced by the SOSE model (fig. S2). SOSE captures the surface salinity over the Taylor cap and the flanks of the Maud Rise to within ±0.02 PSU during the winter season (July through September) in the years 2013 to 2018. The difference is considerably higher (≈−0.5 PSU) during the melt season (December through February) in the 2013–2015 period and reduces during 2016 to 2018 (≈0.2 PSU). Melt season biases have a limited impact on our analysis, which is focused on wintertime destratification. To gain further confidence in SOSE, we compare the SOSE-generated sea ice concentration in September and October 2017 with satellite observations (*22*) in fig. S3. SOSE adequately reproduces the reduced sea ice cover over the northern flank of Maud Rise, where the polynya is initially triggered. However, SOSE has some biases in the spatial extent and magnitude of sea ice concentration anomalies, with a positive bias over the Taylor cap. This indicates that the polynya does not spread as quickly in SOSE, likely due to possible biases in the representation of ocean vertical mixing and sea ice dynamics. Biases have recently been reported in sea ice model thermodynamics and ERA5 prescribed radiative flux estimates in this region (*58*). However the SOSE sea ice biases reduce substantially toward the end of October 2017.

For the Southern Ocean, SOSE is a well-established model that does well at representing the ocean dynamics, thermodynamics (*59*), and sea ice of the Southern Ocean (*60*). For this study, we restrict the model solution to the period from 2013 to 2018. Our study domain was restricted to the red box shown in Fig. 1A, which was selected to encompass the Maud Rise and its northern flank and the downstream Weddell Sea immediately to the southwest of the Rise.

### Salinity budget

Salinity in the model is expressed in grams per kilogram and is equivalent to practical salinity units (*61*). The salinity budget was computed by time-integrating the salinity tendency equation

$$\frac{\partial S}{\partial t}=G_{\text{adv}}+G_{\text{diff}}+G_{\text{surf}}+G_{\text{corr}}$$ (1)

and vertically averaging it in the uppermost 20 m, to identify processes that contributed to surface salinification over the study period. Our focus on salinity stems from its control of upper-ocean stratification in the Maud Rise and wider Weddell Gyre regions (*62*). The budget involves horizontal (Adv_h) and vertical (Adv_v) advection terms (∫*G*_adv_*dt*), horizontal (Diff_h) and vertical (Diff_v) diffusion (∫*G*_diff_*dt*) modeled using an eddy kinetic energy–based parameterization (*63*), net surface fluxes of precipitation, evaporation, runoff, and sea ice are prescribed as virtual salt (and heat) fluxes at the surface (surf; ∫*G*_surf_*dt*), and a small correction due to the nonlinear free surface assumption (∫*G*_corr_*dt*). The spatial average of these terms over a given area directly represents the spatially averaged rate of change of salinity and is referred to as the “salinity balance” through the rest of the text.

The vertically averaged horizontal Ekman advection of salinity was estimated by time-integrating and spatially averaging the following expression

$${\text{Ek}}_{\text{adv}}=\frac{U}{\delta_{\text{Ek}}}\frac{\partial <S{>}_{\text{Ek}}}{\partial x}+\frac{V}{\delta_{\text{Ek}}}\frac{\partial <S{>}_{\text{Ek}}}{\partial y}$$ (2)

where <*S*>_Ek_ is the vertically averaged salinity in the Ekman layer, and the vertically integrated Ekman velocities are defined as **U** = τ*_y_*/(ρ_0_*f*) and **V** = −τ*_x_*/(ρ_0_*f*), with τ*_x_* and τ*_y_* being the zonal and meridional surface stresses, and ρ_0_ = 1035 kg m^−3^. The thickness of the Ekman layer is estimated using δ_Ek_ = 0.4*u*^∗^/∣*f*∣, where $u^{*}=\sqrt{|\tau |/\rho_{0}}$ denotes the frictional velocity and *f* the Coriolis parameter (*64*, *65*). The average Ekman layer depth was 22 m over the Taylor cap of Maud Rise (area shallower than 3000 m; fig. S9), thus informing our choice to compute salinity budgets in the upper 20 m through the rest of the manuscript.

### PV framework

An Ertel PV framework is applied in this analysis to assess the drivers of stratification. PV is defined as *q* = −(ω_a_ ⋅ ∇ σ)/ρ_0_, where $\omega_{a}=\left(\nabla \times \vec{v}+f\hat{k}\right)$  is the absolute vorticity, $\hat{k}$ is the unit vector in the vertical direction, and σ is the potential density at a reference pressure of 0 dbar. The PV budget is computed using the full three-dimensional fields as derived by Marshall and Nurser (*36*). PV is computed for the uppermost 500 m of the water column, in line with this study’s focus on near-surface processes. This depth range encompasses the exchange of salinity and heat between the mixed layer and the WDW beneath the pycnocline (see fig. S8). Consequently, it allows for the utilization of PV as a proxy to examine the evolution of stratification within the upper 500 m of the ocean throughout the study period. The PV conservation equation is

$$\frac{\partial \rho q}{\partial t}+\nabla \cdot \vec{J}=0$$ (3)

where ρ is the density and $\vec{J}$ denotes the PV flux vector

$$\vec{J}=\underset{{\vec{J}}^{A}}{\underbrace{\rho q\vec{v}}}-\underset{{\vec{J}}^{B}}{\underbrace{\frac{1}{g}B\omega_{a}}}+\underset{{\vec{J}}^{F}}{\underbrace{\vec{F}\times \nabla \sigma }}$$ (4)

Here, $B=-g\frac{\mathrm{D\sigma}}{\mathrm{Dt}}$ is the buoyancy forcing, and

$$\vec{F}=\frac{D\vec{v}}{\mathrm{Dt}}+f\hat{k}\times \vec{v}+\frac{1}{\rho_{0}}\nabla p$$ (5)

where $\vec{F}$ is the frictional force (computed using Eq. 5, which is a residual of the momentum equation), and ∇*p* is the pressure gradient. The flux vector $\vec{J}$ contains the advective terms of the PV flux, ${\vec{J}}^{A}$ , and the nonconservative terms associated with boundary layer processes inducing a flux in PV, namely, ${\vec{J}}^{B}$ due to buoyancy fluxes, and **J**^F^ due to surface frictional forces (principally, the wind-driven Ekman lateral force). We note here that only the lateral components of the friction term is considered in this analysis (i.e., ${\vec{J}}^{F}$ is assumed to be oriented in the vertical direction) as its vertical component is much smaller in comparison.

In the main text, the budget terms are shown as time-integrated quantities. For the maps (Fig. 5, A and B), we time integrated the buoyancy and frictional components of PV from March through September each year, with the integration constant set to zero. However, when presenting the entire budget as spatial averages over the northern flank (Fig. 5D), we perform the time integration using an integral constant that matches the initial PV value at time zero.

### Observational data

Observed ocean surface stress was computed as: τ_total_ = (sic) ∗ τ_iw_ + (1 − sic)τ_aw_, where sic is the sea ice concentration, τ_iw_ = ρ_w_*C*_iw_∣*u*_ice_∣*u*_ice_ is the stress between sea ice and water, with seawater density as ρ_w_ = 1035 kg/m^3^, *C*_iw_ = 5.5 × 10^−3^ is the drag coefficient between sea ice and water, and *u*_ice_ is the velocity of sea ice. τ_aw_ = ρ_air_*C*_d_∣*u*_10_∣*u*_10_ is the stress between air and water, ρ_air_ = 1.25 kg/m^3^ is the density of air, *C*_d_ = 1.25 × 10^−3^ is the drag coefficient between air and water, and *u*_10_ is the wind velocity at 10 m elevation above sea level.

Observations in this work are from profiling float (*66*, *67*) and tagged seal (*68*, *69*) datasets (location of profiles plotted in figs. S4 and S5; tag numbers provided in section S1). The observed salinity was spatially averaged over the region of study displayed in Fig. 2A. We caution that the observations have spatiotemporal sampling biases, such that here they are used solely to provide context to the SOSE estimates.

The seal tag dataset goes through a “delayed mode” calibration process (*70*, *71*). The process corrects for pressure effects on salinity and temperature, thermal mass errors, salinity spiking, and density inversions. Further, field effects on the conductivity sensor cause salinity offsets. These are corrected for using the stable salinity and temperature in the lower CDW layer sampled by the seals that forage close to the Antarctic continent. If this is not available, statistical cross-correlation techniques are used using tags that measure nearby. Only the data that went through this process and were flagged as good quality were used in the assimilation. Siegelman *et al.* (*71*) estimate the accuracy of the tagged seals dataset after calibration at ±0.04°C and ±0.03 g/kg for temperature and salinity, respectively.

### Additional methodological considerations

The overturning limit of salinity is defined as the salinity at which the mixed-layer potential density becomes equal to the potential density at a depth of 260 m (a depth that lies below the pycnocline and within the WDW layer). The term Δ_ov_ is defined as the difference between the mixed-layer salinity and the overturning limit of salinity. This provides an upper-bound estimate of the salinity required to push the water column into an overturning regime and is known as the salt deficit (*37*).

Mixed-layer depth in SOSE was diagnosed using a density gradient test, which identifies the depth at which the maximum second derivative of density occurs $\left(\frac{\partial^{2}\sigma^{\theta }}{\partial z^{2}}\right)$ above the depth at which the maximum first derivative of density occurs $\left(\frac{\partial \sigma^{\theta }}{\partial z}\right)$ . This algorithm is subject to certain checks as detailed in Chen *et al.* (*72*).

Time series plots of budget terms typically include monthly climatological means and their deviations. These monthly means, calculated as averages for each month across all years, are represented by broken lines. Five-day averaged values are depicted with continuous lines, with shading used to emphasize the difference between these instantaneous values and the corresponding monthly means. Note that some budget terms show an interannual trend when the climatological monthly mean values are time integrated. This is to be expected, for instance, in the case of surface flux of salinity and vertical diffusion of salinity, as the region is a net importer of sea ice that forms in coastal polynyas and is advected to this region.

Throughout this article, the area shallower than 3000 m over the Maud Rise is referred to as the Taylor cap. In turn, the region to the north of the Maud Rise, bounded by the 3000- and 5000-m isobaths and by longitudes of 2°E and 5.5°E, is referred to as the “northern flank” (see Fig. 2). The northern flank was chosen to capture the jet flowing on the northern periphery of the seamount where the polynya was initially triggered.

## Supplementary Materials

### This PDF file includes:

Figs. S1 to S12 Section S1
