# Supplementary material for: Ekman-driven salt transport as a key mechanism for open-ocean polynya formation at Maud Rise
Source: Sci Adv. 2024 May 1;10(18):eadj0777. doi: 10.1126/sciadv.adj0777 (PMC11062571; doi:10.1126/sciadv.adj0777)
Supplement: Supplementary file 1 — Figs. S1 to S12 Section S1 [file sciadv.adj0777_sm.pdf]

Science Advances
AAAS

Supplementary Materials for

Ekman-driven salt transport as a key mechanism for open-ocean polynya formation at Maud Rise

Aditya Narayanan *et al.*

Corresponding author: Aditya Narayanan, a.narayanan@soton.ac.uk

Sci. Adv. **10**, eadj0777 (2024)
DOI: 10.1126/sciadv.adj0777

This PDF file includes:

Figs. S1 to S12
Section S1

1 List of platform identifiers for the float and seal tags

used in Figure 2

The platform identifiers listed below are the World Ocean Database identifiers:

7900071, 7900224, 5901735, 6900589, 5901720, 7900341, 5903616, 5903615, 5904468, 5904471, 5904467, 7900074, 7900072, 7900040, 7900060, 5901734, 5901743, 5901729, 7900226, 5905380, 7900073, 5901727, 5904478, 5904397, 5901737, 5903613, 5903614, 5903709, 5905381, 7900041, 7900122, 5901723, 7900343, 1901906, 7900076, 7900227, 7900340, 7900289, 5905383, 1901903, 6903256, 7900123, 6903255, 5901718, 5901731, 6900587, 5904472, 1901904, 6900584, 1901251, 1902079, 7900231, 7900126, 7900042, 5905382, 00058710, 00058854, 00033498, 00014718, 00051906, 00013770, 00013734, 00013806, 00013764, 00013752, 00013668, 00025410, 00014652, 00014646

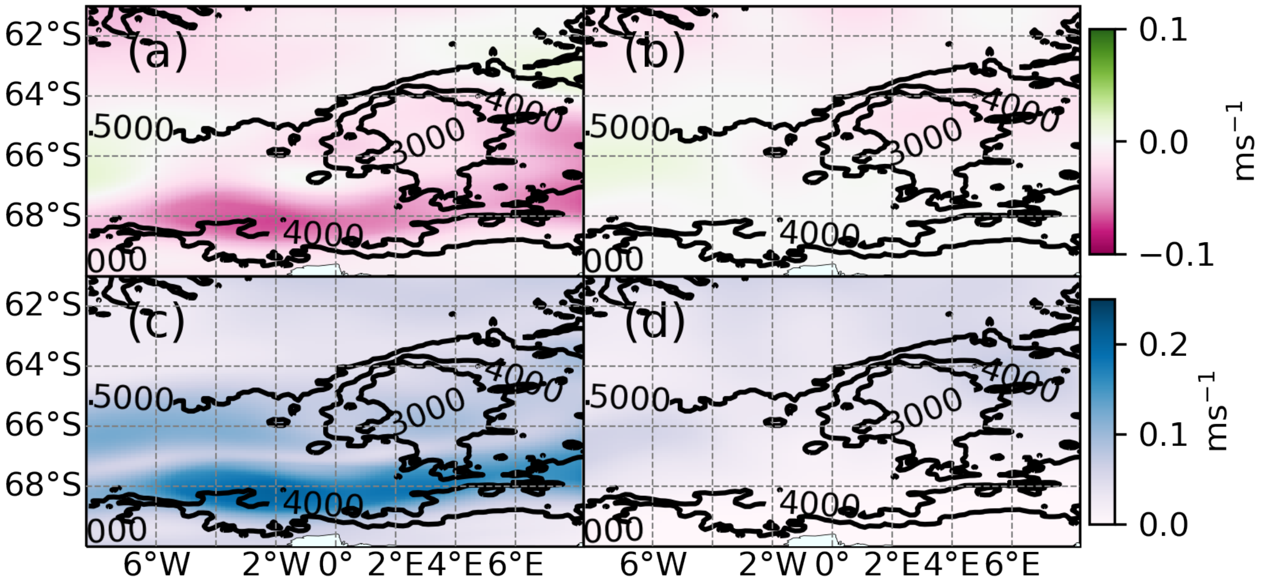

Supplementary Figure 1: Mean of the adjustments to: **a.** zonal wind speed, and **b.** meridional wind speed. Standard deviation of the adjustment to: **c.** zonal wind speed, and **d.** meridional wind speed. Note the factor of 5 difference in color scale ranges.

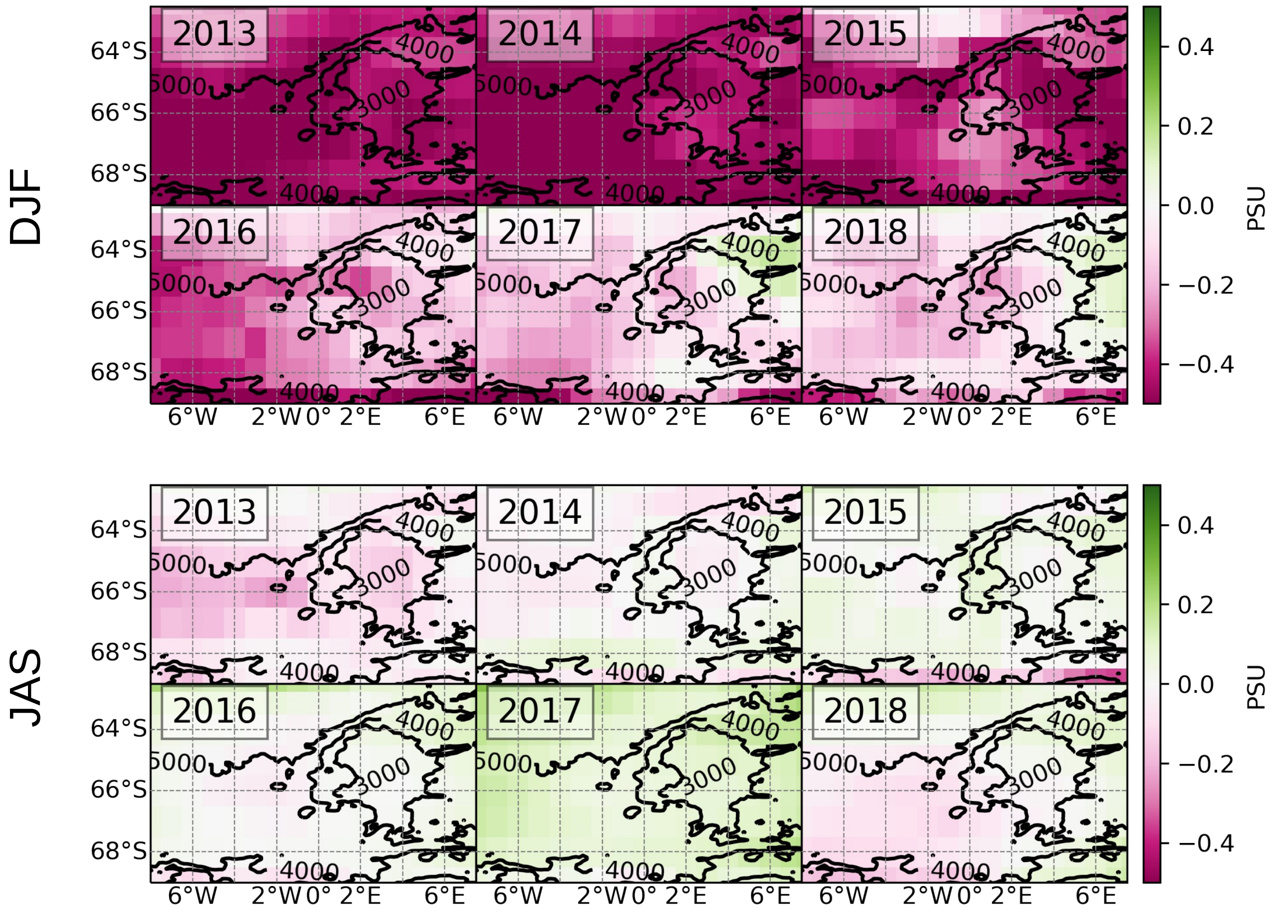

Supplementary Figure 2: SOSE minus EN4.2 near-surface salinity (upper 20 m) for the seasons December-February (DJF) and July-September (JAS).

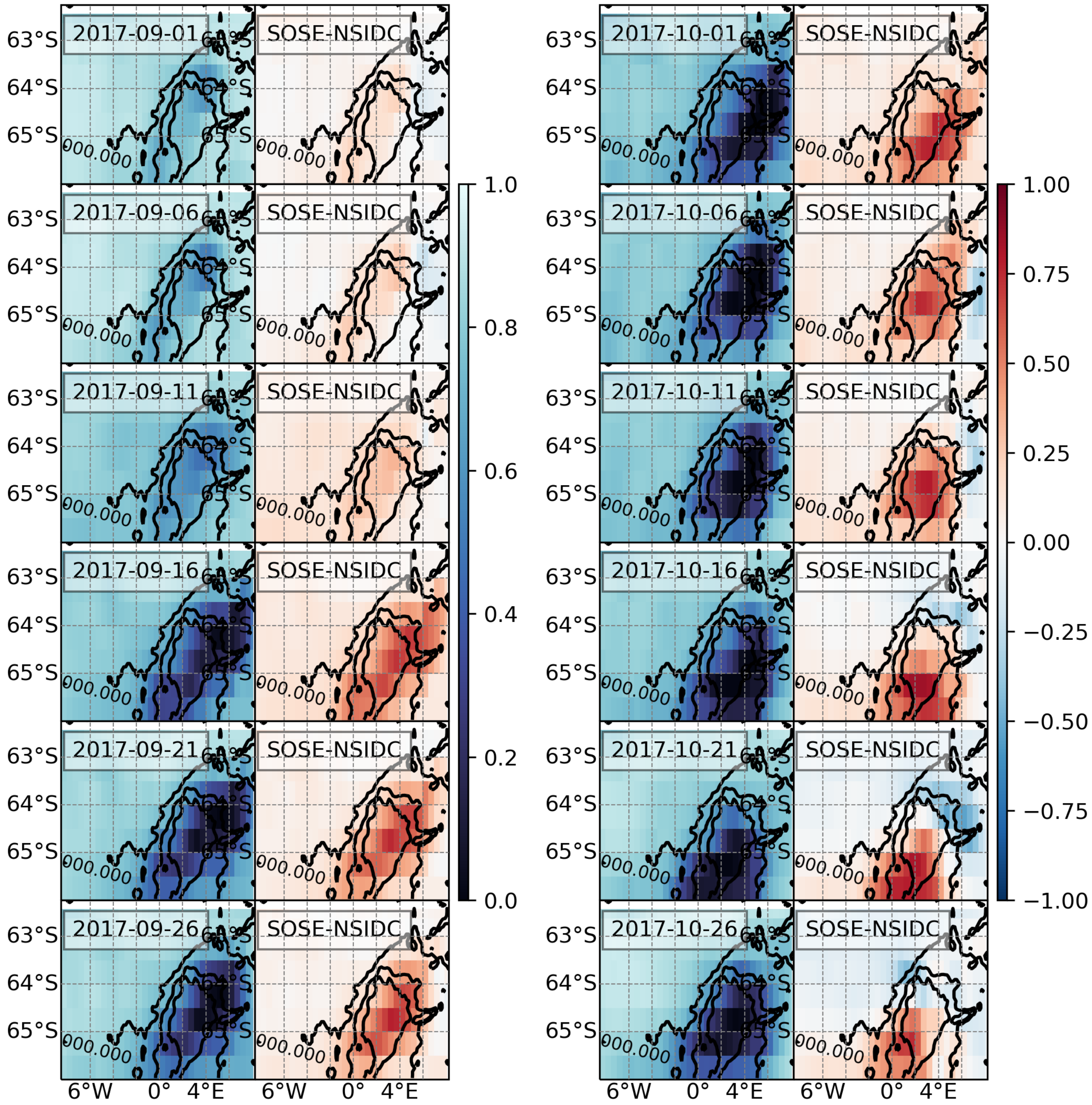

Supplementary Figure 3: Remotely sensed sea ice concentration (from NSIDC) in September and October 2017, and biases in SOSE with respect to NSIDC. NSIDC sea ice concentration has been binned in time to match the 5-day averaged output in SOSE. SOSE data have been subsampled on the spatial grid of NSIDC.

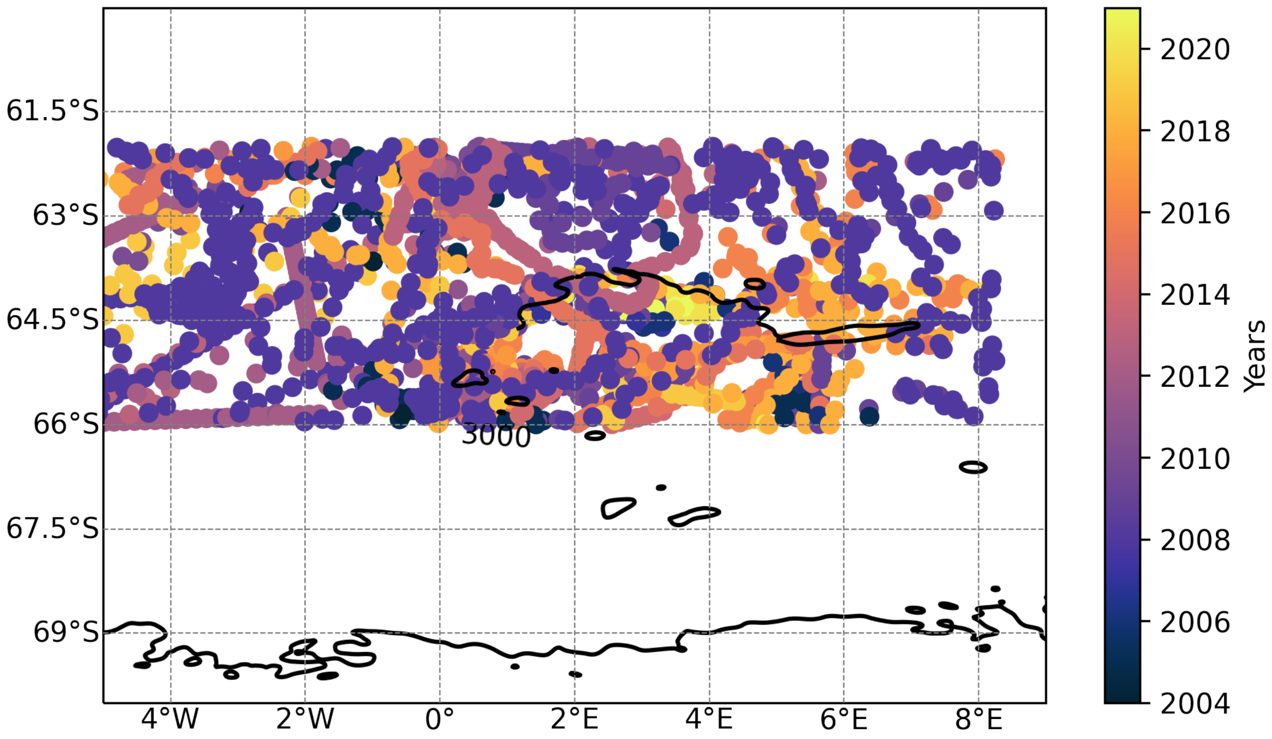

Supplementary Figure 4: Locations of profiles over Maud Rise used in Figure 2.

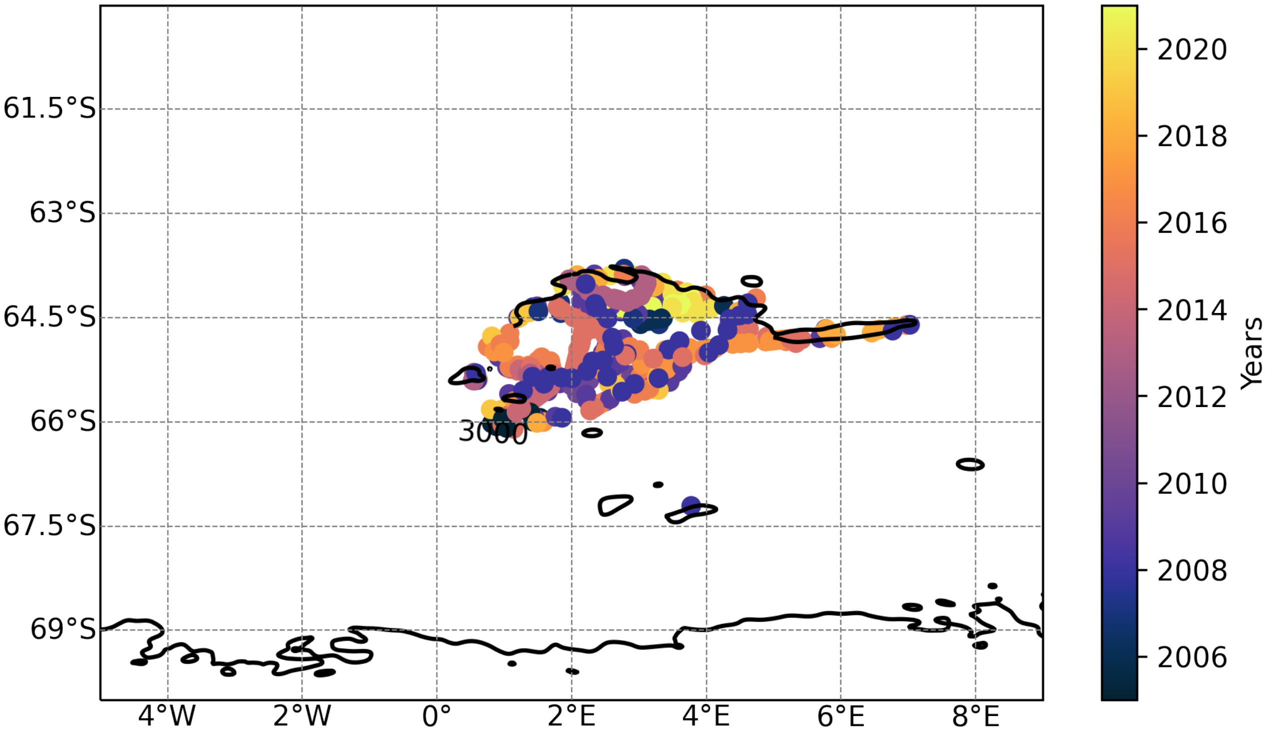

Supplementary Figure 5: Locations of profiles over the Taylor Cap of Maud Rise used in Figure 8.

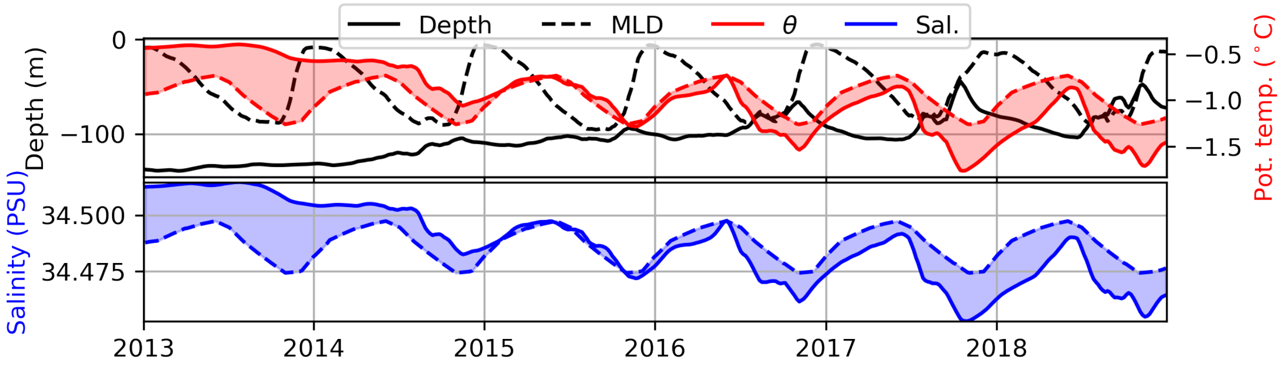

Supplementary Figure 6: Mixed layer depth (broken black line), depth of the 27.74 kg m^{-3} isopycnal (solid black line) and its corresponding temperature (red) and salinity (blue). Shaded red and blue areas are anomalies with respect to the monthly means (broken lines).

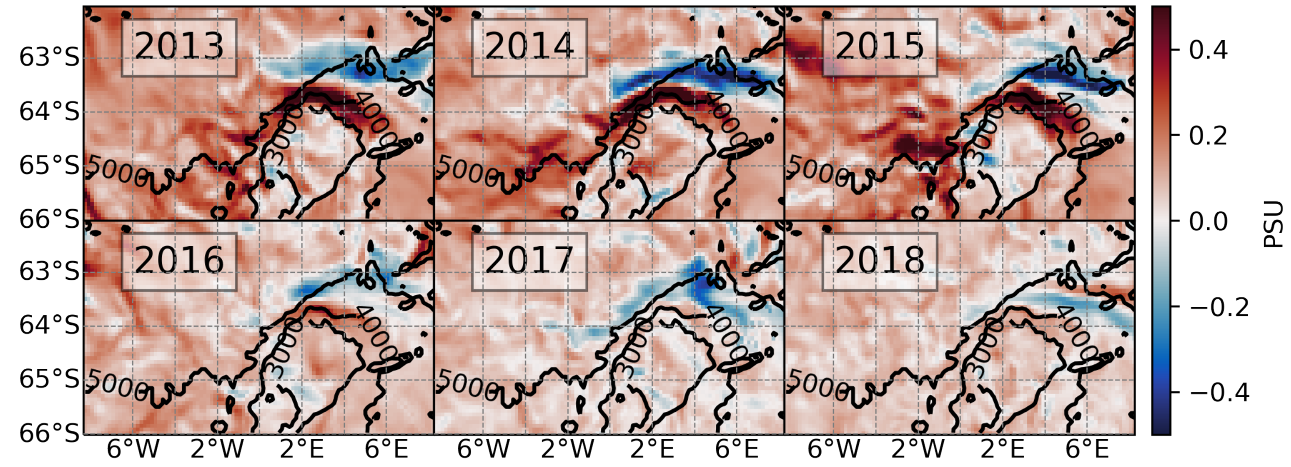

Supplementary Figure 7: Sum of the surface salinity flux and the vertical diffusion of salinity, vertically averaged in the upper 20 m and time-integrated from March to September for the years 2013 to 2018.

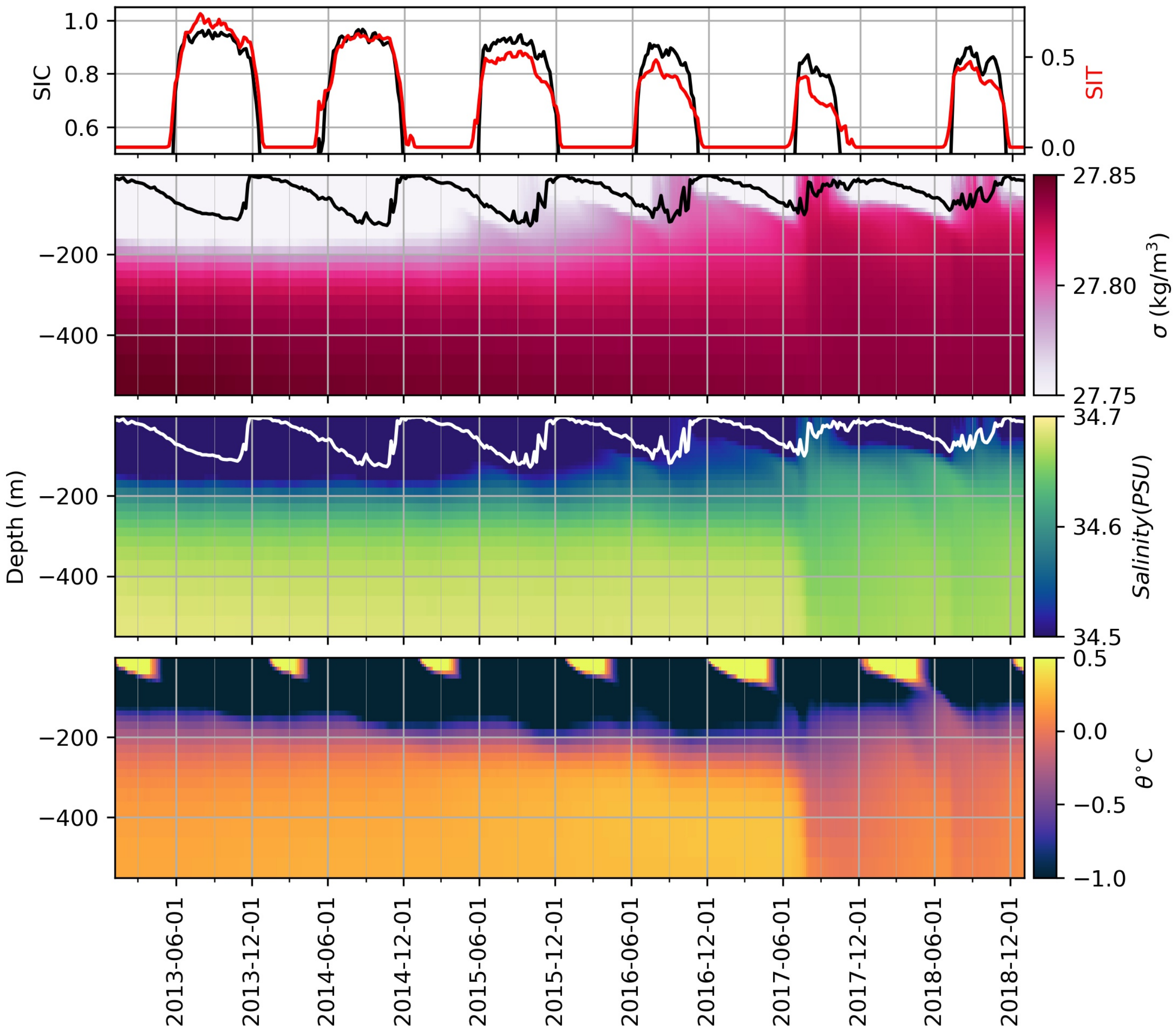

Supplementary Figure 8: SOSE-derived: Hydrography and sea ice, spatially averaged over the Taylor Cap: **a.** Sea ice concentration and thickness, **b.** potential density (black line is the mixed-layer depth), **c.** salinity (white line is the mixed-layer depth), **d.** potential temperature.

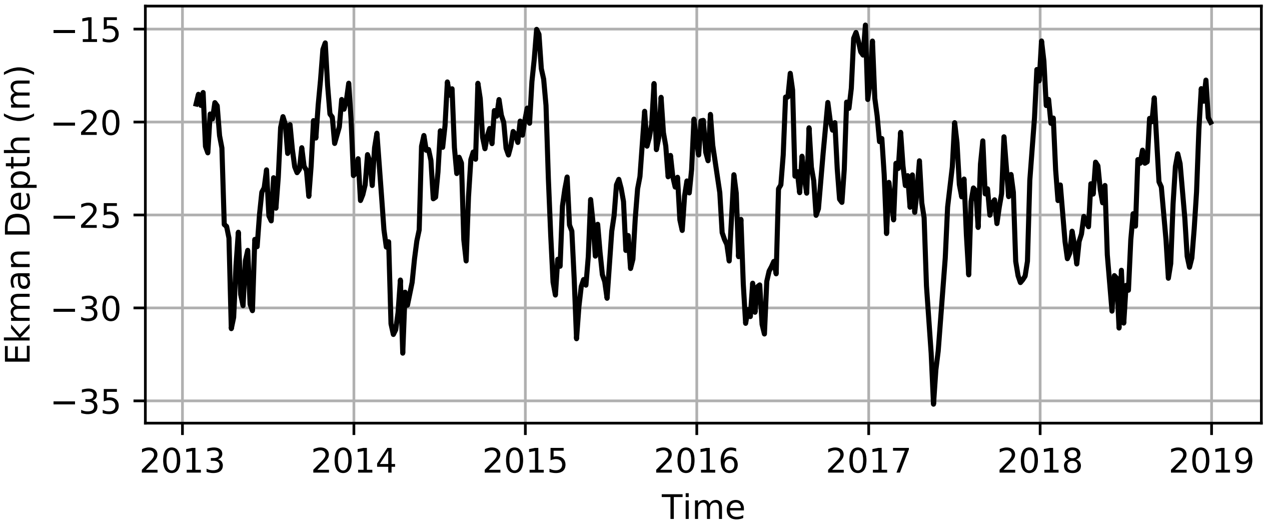

Supplementary Figure 9: Ekman layer depth, spatially averaged over the Taylor Cap and the northern flank of Maud Rise.

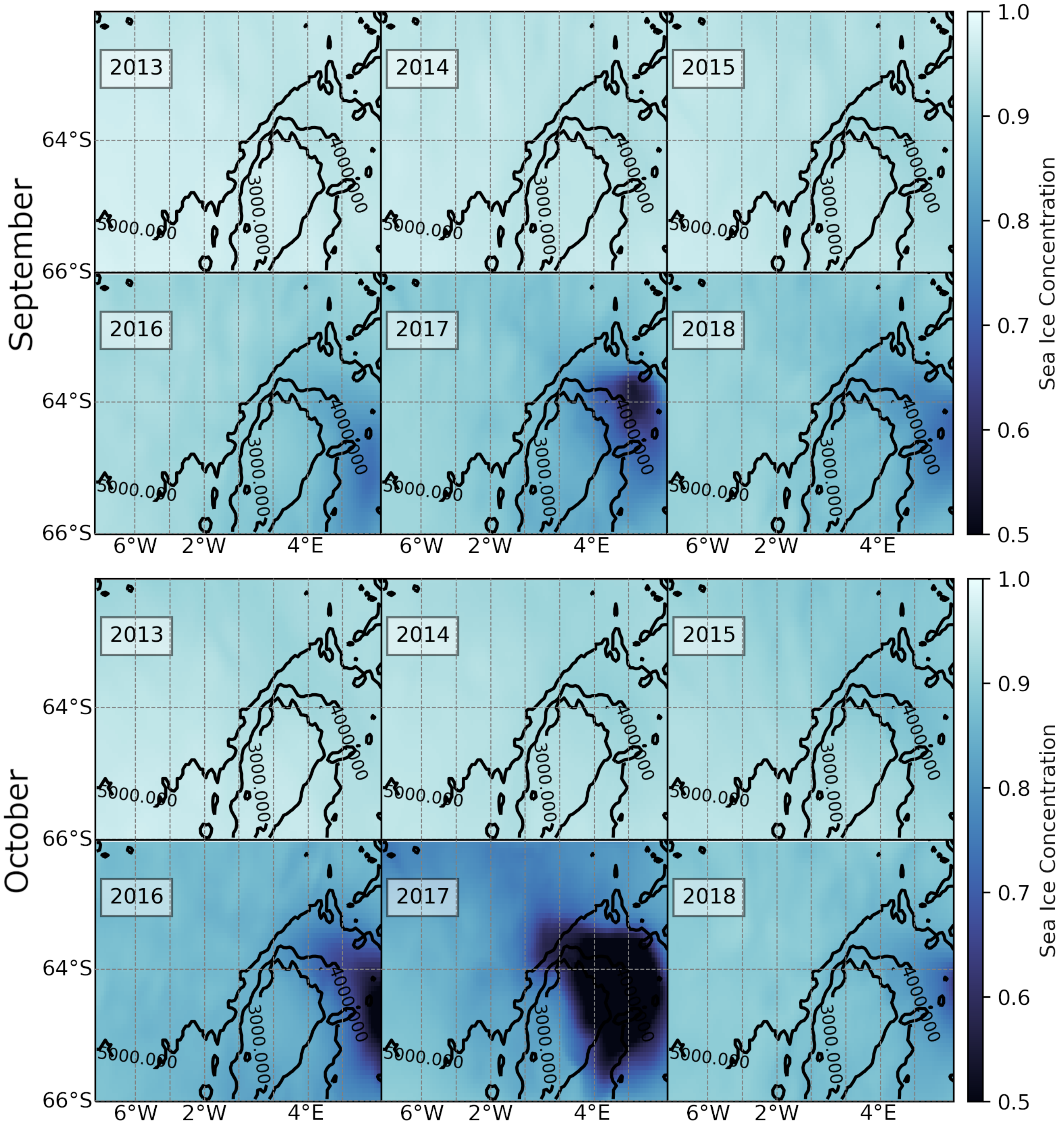

Supplementary Figure 10: Sea ice concentration from SOSE averaged over the months of September and October for each year from 2013 to 2018.

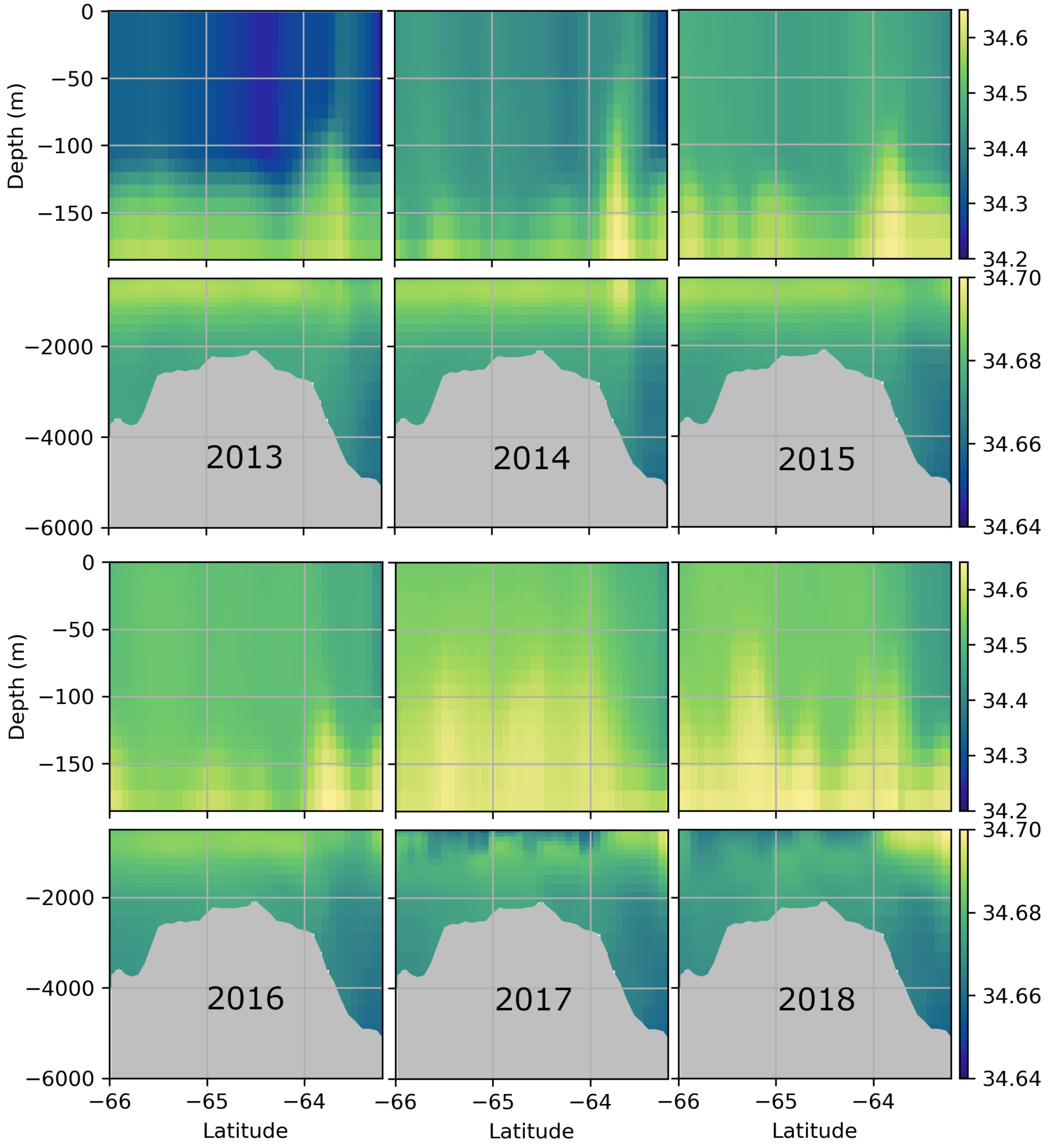

Supplementary Figure 11: Salinity along 3°E across Maud Rise for each year, averaged over September. Note that the scale of the y-axis and that of the color map vary between the surface panels and the sub-surface panels.

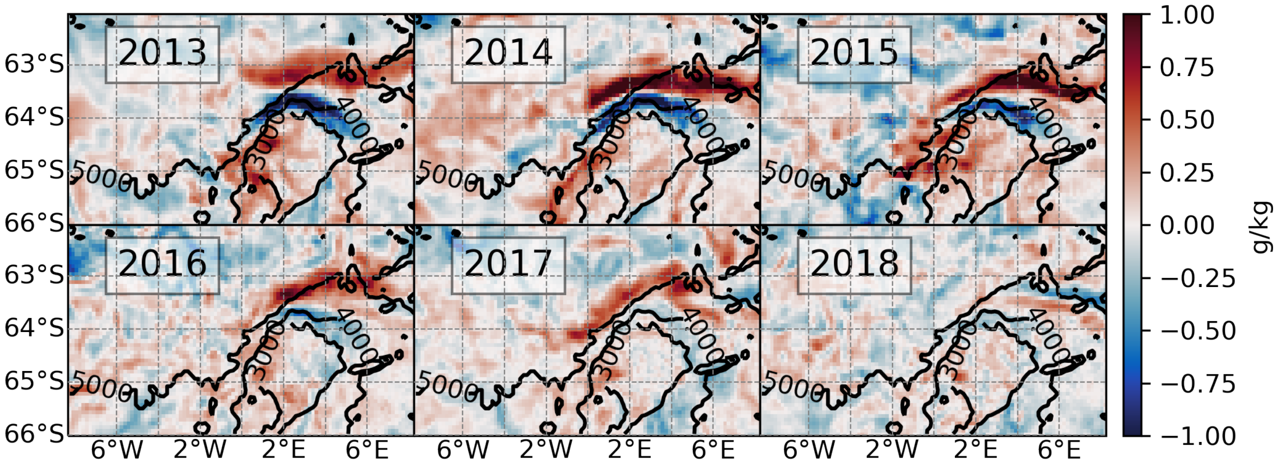

Supplementary Figure 12: The divergence of the horizontal advection of salinity (G_{adv} in Equation 1, Methods) vertically averaged in the upper 20-m layer and time integrated from March through September of each year.
